# Racial and ethnic disparities in obstetric anal sphincter injury: cross‐sectional study in the USA

**DOI:** 10.1002/uog.29231

**Published:** 2025-05-09

**Authors:** M. Rajasingham, P. Hossein‐Pour, R. D'Souza, R. Geoffrion, C. V. Ananth, G. M. Muraca

**Affiliations:** ^1^ Department of Obstetrics and Gynecology, Faculty of Health Sciences McMaster University Hamilton ON Canada; ^2^ Department of Health Research Methods, Evidence and Impact, Faculty of Health Sciences McMaster University Hamilton ON Canada; ^3^ Department of Obstetrics and Gynaecology University of British Columbia Vancouver BC Canada; ^4^ Division of Epidemiology and Biostatistics, Department of Obstetrics, Gynecology, and Reproductive Sciences Rutgers Robert Wood Johnson Medical School New Brunswick NJ USA; ^5^ Cardiovascular Institute of New Jersey Rutgers Robert Wood Johnson Medical School New Brunswick NJ USA; ^6^ Department of Medicine Rutgers Robert Wood Johnson Medical School New Brunswick NJ USA; ^7^ Clinical Epidemiology Unit, Department of Medicine Solna, Karolinska Institutet, Eugeniahemmet Stockholm Sweden

**Keywords:** ethnic disparities, fourth‐degree perineal lacerations, health equity, immigrant maternal health, OASI, obstetrical anal sphincter injury, obstetrical trauma, racial disparities, third‐degree perineal lacerations

## Abstract

**Objectives:**

Racial disparities in obstetric anal sphincter injury (OASI) are poorly understood; their investigation by parity, obstetric history and mode of delivery may provide insight into which individuals are at the greatest risk for OASI. We aimed to quantify the association of race and ethnicity with OASI, stratified by parity, obstetric history and mode of delivery. Secondary aims were to explore variations in OASI rates among racial subgroups and by immigration status (foreign‐born *vs* USA‐born).

**Methods:**

We conducted a cross‐sectional study of 12 501 183 vaginal births in the USA from January 2016 to December 2021 using birth‐certificate data obtained from the National Vital Statistics System. Cox proportional hazard regression models were fitted, with gestational age as the timescale, to quantify the association of self‐reported race and ethnicity with OASI, with adjustment for several confounders. The maternal race and ethnicity groups included: American Indian or Alaska Native (AIAN), Asian, Black, Hispanic, Native Hawaiian and other Pacific Islander, White and mixed race. Models were stratified by number of previous births and the occurrence of Cesarean delivery (CD) among prior births. This resulted in three groups: primiparous (i.e. only the index birth); multiparous without a previous CD; and multiparous with at least one previous CD. Within each stratum, we further grouped individuals by mode of delivery in the index birth, as spontaneous vaginal delivery (SVD), operative vaginal delivery (OVD) with forceps and OVD with vacuum.

**Results:**

In primiparous individuals who had a vaginal birth, the overall OASI rate was 2.2%, but it varied widely by mode of delivery (SVD, 1.7%; OVD with forceps, 14.8%; OVD with vacuum, 6.6%). Asian primiparae had higher OASI hazards compared with White primiparae, irrespective of mode of delivery (SVD: adjusted hazard ratio (aHR), 1.69 (95% CI, 1.64–1.73); OVD with forceps: aHR, 1.48 (95% CI, 1.38–1.58); OVD with vacuum: aHR, 1.51 (95% CI, 1.44–1.58)), while AIAN and Black primiparae had inconsistent associations with OASI rate depending on mode of delivery, when compared with White primiparae. In multiparous individuals without a previous CD, the rates of OASI were lower than those seen in primiparae (SVD, 0.5%; OVD with forceps, 7.5%; OVD with vacuum, 3.2%) and the association of race and ethnicity with OASI varied by mode of delivery for all race groups except Asian, in whom it was consistently associated with a 1.5–2.1‐times higher hazard of OASI. Among multiparous individuals with a previous CD, overall OASI rates were similar to those seen in primiparae (SVD, 1.3%; OVD with forceps, 11.8%; OVD with vacuum, 5.1%). In this group, the only associations of race and ethnicity with OASI were higher hazards among Asian *vs* White individuals who had a SVD (aHR, 2.16 (95% CI, 1.97–2.36)) and an OVD with vacuum (aHR, 1.65 (95% CI, 1.39–1.96)). The rate of OASI varied widely between Asian race subgroups, with the highest rate noted among individuals with origins and/or ancestry from India (e.g. 27.2% among primiparae who had OVD with forceps) and the lowest in those from Japan (e.g. 9.3% among primiparae who had OVD with forceps). Across racial and ethnic minority groups, the OASI rate was higher among foreign‐born *v*s USA‐born residents; this trend was not observed among White individuals.

**Conclusions:**

Racial and ethnic disparities in OASI persist regardless of parity, obstetric history and mode of delivery. Variations in the OASI rate are apparent within Asian racial subgroups and by immigration status. © 2025 The Author(s). *Ultrasound in Obstetrics & Gynecology* published by John Wiley & Sons Ltd on behalf of International Society of Ultrasound in Obstetrics and Gynecology.

## INTRODUCTION

Obstetric anal sphincter injury (OASI) is a serious yet understudied complication of vaginal birth that affects 4.4% of birthing individuals in the USA^1^. OASI is defined as a third‐ or fourth‐degree perineal laceration that involves a separation injury of the external and internal anal sphincter complex^2^. While this injury is associated with considerable short‐term morbidity, including infection and other wound complications, our understanding of its long‐term consequences has evolved to now include the risk of anal incontinence, adverse effects on postpartum mental health and recurrent injury to the anal sphincter^2^, ^3^. Racial and ethnic disparities in OASI are prevalent among Western countries^4^, ^5^, ^6^, ^7^. For instance, in the USA, the rate of OASI is 9.3% among Asian individuals, 2.7% among Black individuals and 4.2% among White individuals^8^. Although several hypotheses have been tested, no compelling explanation exists for these racial and ethnic disparities^7^.

A higher OASI rate has been noted among nulliparous individuals and among individuals who have had at least one previous Cesarean delivery (CD) followed by a vaginal delivery^2^, ^4^, ^9^, ^10^. The OASI rate also varies substantially according to the mode of delivery, with the highest rate observed in operative vaginal delivery (OVD) with forceps, followed by OVD with vacuum and spontaneous vaginal delivery (SVD)^2^, ^3^, ^11^. Given that parity, obstetric history and mode of delivery are strong determinants of the risk of OASI, evaluating the association of race and ethnicity with OASI in groups defined by these determinants may provide novel insights into the established racial disparities. Thus, this study aimed to assess racial and ethnic disparities in OASI, stratified by mode of delivery, among USA residents who were (1) primiparous, (2) multiparous without previous CD or (3) multiparous with at least one previous CD. We hypothesized that OASI is differentially distributed by race and ethnicity among all study strata, and that the strength of these associations varies by mode of delivery, parity and obstetric history. Secondary aims of this study included an exploration of the OASI rate among racial subgroups and by immigration status.

## METHODS

### Study population

We conducted a population‐based cross‐sectional study of all live singleton births between January 2016 and December 2021 in the USA, using the natality data files from the National Vital Statistics System (NVSS). NVSS collects data annually on approximately 99% of all registered births within the 50 USA states and the District of Columbia^12^. Data were extracted from birth certificates, which combine information from two standardized worksheets: the ‘Facility Worksheet’ (including data from medical records) and the ‘Mother's Worksheet’ (including self‐reported information)^12^. Data obtained from the files consisted of maternal characteristics (e.g. age, race, ethnicity, prepregnancy body mass index (BMI)), comorbidity indicators (e.g. gestational diabetes mellitus, pre‐eclampsia) and obstetric practice factors (e.g. mode of delivery, induction, epidural anesthesia).

Our analysis was confined to the years 2016 to 2021. Coding inconsistencies between years were addressed by recoding variables to match 2021 encodings. This study followed the Strengthening the Reporting of Observational Studies in Epidemiology (STROBE) guidelines and the race and ethnicity reporting guidelines outlined by the American Medical Association^13^.

### Study variables

We operationalized the term ‘race’ as a social construct by which individuals are categorized based on perceived physical differences, and ‘ethnicity’ as cultural factors (i.e. language, religion, nationality)^14^. In this study, race and ethnicity were employed jointly as a proxy measure for systemic racism, the study's exposure, in accordance with the theory of intersectionality and the Critical Race Theory^14^, ^15^, ^16^, ^17^. Maternal race and ethnicity were self‐reported via two distinct questions in the ‘Mother's Worksheet’, regarding self‐identified race and Hispanic origin. NVSS categorized race and ethnicity into seven groups: (1) non‐Hispanic American Indian or Alaska Native (AIAN); (2) non‐Hispanic Asian; (3) non‐Hispanic Black; (4) Hispanic; (5) non‐Hispanic Native Hawaiian and other Pacific Islander (NHOPI); (6) non‐Hispanic White; and (7) non‐Hispanic mixed race^12^. We refer to these racial groups as AIAN, Asian, Black, Hispanic, NHOPI, White and mixed, respectively. When self‐reported maternal race was unavailable, race was assigned by NVSS based on paternal race or a previous birth‐certificate record with known maternal race, if one existed^12^.

The outcome was OASI, defined as a third‐ or fourth‐degree perineal laceration^18^. Third‐degree perineal laceration refers to that which extends through the perineal skin, vaginal mucosa, perineal body and the internal and/or external anal sphincter, while a fourth‐degree laceration includes additional extension through the rectal mucosa^3^. NVSS extracted OASI diagnosis data from delivery‐ and recovery‐room records^18^.

The following covariates were included in our adjusted models based on clinical expertise and the previous literature: maternal age (< 19 years, 19–24 years, 30–34 years, 35–40 years, > 40 years *vs* 25–29 years); marital status (married *vs* unmarried); highest level of education (8^th^ grade or below, high school (no degree), high school degree or general educational development, college (no degree), Associate degree, Bachelor's degree, Master's degree *vs* Doctorate degree); payer (Medicaid, self‐pay, other *vs* private insurance); Women, Infants and Children food program; prepregnancy BMI (underweight, < 18.5 kg/m^2^; overweight, 25.0–29.9 kg/m^2^; obese, ≥ 30 kg/m^2^ *vs* normal, 18.5–24.9 kg/m^2^); assisted reproductive technology; prepregnancy diabetes mellitus; prepregnancy hypertension; gestational diabetes mellitus; pre‐eclampsia/eclampsia; induction of labor; augmentation of labor; and birth weight ≥ 4000 g.

### Statistical analysis

Individuals were grouped into three strata based on parity and obstetric history: primiparous (one birth, which is the index birth), multiparous without previous CD (two or more births, all of which were delivered vaginally) and multiparous with at least one previous CD (two or more births, including at least one CD) (Figure S1). We did not include individuals who had a Cesarean delivery in the index birth in our analysis, and stratified the remaining individuals by mode of delivery in the index vaginal birth (SVD, OVD with forceps or OVD with vacuum). We estimated adjusted hazard ratios (aHRs) with 95% CIs using Cox proportional hazard regression models, with gestational age at delivery as the timescale, to quantify the association of race and ethnicity with OASI. This approach was chosen to satisfy two considerations: (1) the heightened risk of OASI with increased gestational age and (2) the varying preterm birth rate across racial and ethnic groups^4^, ^19^. Thus, by using Cox proportional hazard regression models, we could compare the risk of OASI across races and ethnicities while accounting for the varying at‐risk period between the groups. Each race and ethnicity group was compared with White individuals, as previous studies have reported the OASI rate in this group to be similar to that in the overall population^5^, ^9^, ^20^. Additionally, we calculated absolute population attributable fractions (PAFs) to estimate the proportion of OASI cases that could have been avoided if the differences by race had been eliminated^21^. A complete‐case approach was followed, as all variables had less than 4% of cases with missing data, except for marital status (9.8% missing data).

Statistical analysis was conducted using SAS version 9.4 (SAS Institute, Cary, NC, USA) and RStudio version 4.3.0 (R Foundation, Vienna, Austria) software with the extension packages ‘episensr’, ‘mice’ and ‘survival’^23^, ^24^, ^25^, ^26^. All analysis was performed on publicly accessible, anonymized data and thus we did not require ethical approval.

### Sensitivity analysis

Among individuals who had had at least one previous CD, data constraints precluded our ability to distinguish whether the prior CD occurred in the birth immediately before the index birth, or if vaginal birth(s) had occurred between the previous CD and the index birth. This caused a potential for bias among the multiparous with at least one previous CD group, as we could not account for differences in OASI risk between individuals who were vaginally primiparous (one vaginal birth that was the index birth) and individuals who had had multiple vaginal births between a previous CD and the index birth. To address this, we conducted a sensitivity analysis restricted to multiparous individuals with at least one previous CD and only one prior birth (i.e. their only previous birth prior to the index birth was by CD) (Figure S1).

**Figure 1:**
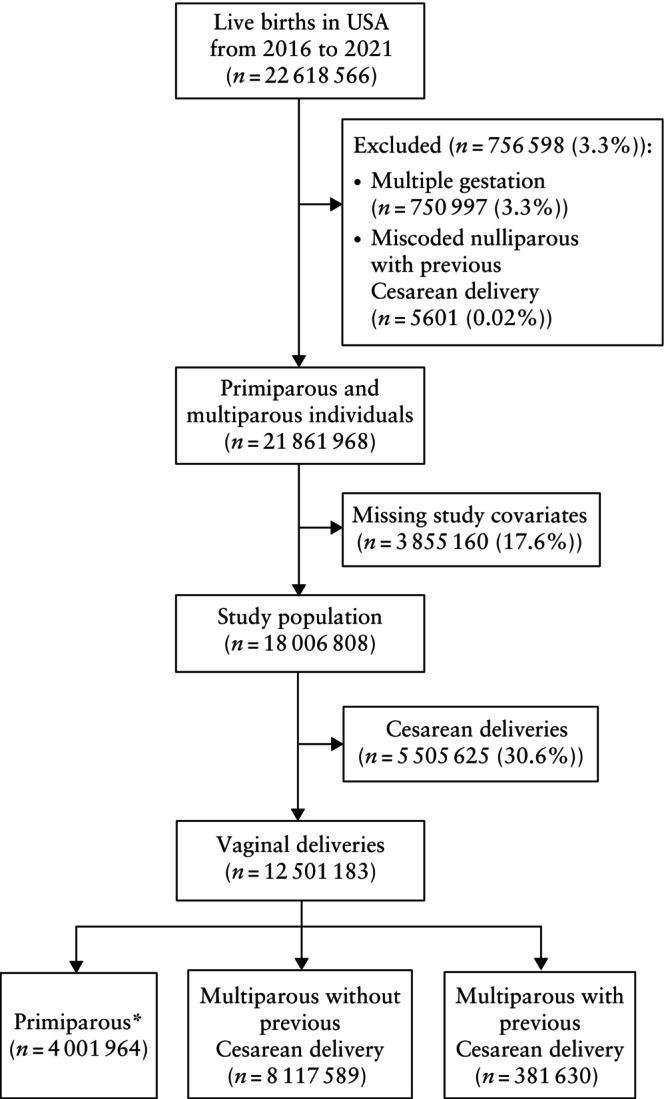
Flowchart summarizing derivation of study cohort. *One birth, which is the index birth.

Additionally, we conducted a series of sensitivity analyses to assess potential bias due to missing data, outcome misclassification, unmeasured confounding and heterogeneity in our racial and ethnic groups. First, missing values for study variables were imputed using multiple imputation and the results from 10 multiple imputation cycles were combined using PROC MIANALYZE in SAS. Second, to assess the impact of the OASI indicator's sensitivity^22^, we conducted a probabilistic bias analysis exploring outcome misclassification and unmeasured confounding. Third, we assessed heterogeneity in the risk of OASI among Asian racial and ethnic subgroups (self‐identified groupings of Chinese, Filipino, Indian, Japanese, Korean, Vietnamese and other Asian)^12^, and within all racial and ethnic groups by place of birth (foreign‐born *vs* USA‐born).

## RESULTS

There were 22 618 566 live births in the USA between 2016 and 2021, of which 756 598 (3.3%) were excluded from the study because of multiple gestation and/or unclear parity information. We further excluded 3 855 160 (17.6%) births owing to missing information regarding any or all the study covariates. Thus, 18 006 808 births were included in our study (Figure 1). Characteristics of individuals with missing information can be found in Table S1. Of the study population, 54.5% of individuals were White, 21.8% were Hispanic, 15.0% were Black and 5.5% were Asian. AIAN, NHOPI and mixed‐race individuals comprised 3.3% of the population (Table 1). A higher proportion of Asian individuals were 35 years and older, conceived by assisted reproductive technology and had a lower rate of pre‐eclampsia/eclampsia. AIAN and NHOPI populations had a higher proportion of multiparous individuals with two or more births and tended to have a higher prepregnancy BMI, while Black individuals had a higher rate of prepregnancy hypertension.

**Table 1:** Demographic and clinical characteristics of 18 006 808 individuals who had a live singleton birth in the USA from 2016–2021, according to race and ethnicity

Parameter	AIAN (*n* = 150 312)	Asian (*n* = 987 023)	Black (*n* = 2 692 887)	Hispanic (*n* = 3 919 467)	NHOPI (*n* = 40 486)	White (*n* = 9 819 794)	Mixed race (*n* = 396 839)
Patient demographics							
Age							
≤ 19 years	13 978 (9.3)	6731 (0.7)	189 870 (7.1)	303 889 (7.8)	2278 (5.6)	332 085 (3.4)	33 009 (8.3)
20–24 years	42 187 (28.1)	65 331 (6.6)	678 463 (25.2)	955 329 (24.4)	10 714 (26.5)	1 666 688 (17.0)	109 310 (27.5)
25–29 years	45 315 (30.1)	245 399 (24.9)	797 320 (29.6)	1 121 192 (28.6)	12 271 (30.3)	2 910 211 (29.6)	114 836 (28.9)
30–34 years	31 370 (20.9)	397 303 (40.3)	614 342 (22.8)	909 510 (23.2)	9447 (23.3)	3 140 573 (32.0)	87 508 (22.1)
35–40 years	14 442 (9.6)	222 744 (22.6)	326 965 (12.1)	499 367 (12.7)	4625 (11.4)	1 483 144 (15.1)	42 831 (10.8)
> 40 years	3020 (2.0)	49 515 (5.0)	85 927 (3.2)	130 180 (3.3)	1151 (2.8)	287 093 (2.9)	9345 (2.4)
Married	46 778 (31.1)	870 584 (88.2)	813 159 (30.2)	1 857 413 (47.4)	19 594 (48.4)	7 023 900 (71.5)	179 081 (45.1)
Highest level of education							
8^th^ grade or below	1963 (1.3)	27 093 (2.7)	40 310 (1.5)	343 556 (8.8)	1178 (2.9)	131 751 (1.3)	4140 (1.0)
High school (no degree)	29 265 (19.5)	46 608 (4.7)	306 452 (11.4)	698 556 (17.8)	8623 (21.3)	558 177 (5.7)	44 944 (11.3)
High school degree or GED	55 753 (37.1)	123 728 (12.5)	971 299 (36.1)	1 286 254 (32.8)	15 145 (37.4)	2 108 332 (21.5)	111 638 (28.1)
College (no degree)	38 155 (25.4)	97 852 (9.9)	675 200 (25.1)	750 306 (19.1)	9301 (23.0)	1 876 296 (19.1)	106 068 (26.7)
Associate degree	11 378 (7.6)	60 339 (6.1)	212 463 (7.9)	262 758 (6.7)	2763 (6.8)	955 287 (9.7)	33 104 (8.3)
Bachelor's degree	9965 (6.6)	332 538 (33.7)	319 003 (11.8)	411 540 (10.5)	2687 (6.6)	2 621 690 (26.7)	62 714 (15.8)
Master's degree	3042 (2.0)	216 377 (21.9)	137 906 (5.1)	131 292 (3.3)	655 (1.6)	1 228 196 (12.5)	25 691 (6.5)
Doctorate degree	791 (0.5)	82 488 (8.4)	30 254 (1.1)	35 205 (0.9)	134 (0.3)	340 065 (3.5)	8540 (2.2)
Payer							
Medicaid	100 668 (67.0)	254 559 (25.8)	1 758 687 (65.3)	2 313 332 (59.0)	23 822 (58.8)	2 918 178 (29.7)	199 184 (50.2)
Private insurance	30 831 (20.5)	671 514 (68.0)	778 242 (28.9)	1 136 329 (29.0)	10 513 (26.0)	6 262 869 (63.8)	167 107 (42.1)
Self‐pay	2694 (1.8)	33 952 (3.4)	72 367 (2.7)	311 598 (8.0)	2653 (6.6)	312 246 (3.2)	8625 (2.2)
Other	16 119 (10.7)	26 998 (2.7)	83 591 (3.1)	158 208 (4.0)	3498 (8.6)	326 501 (3.3)	21 923 (5.5)
Enrolled in WIC food program	80 223 (53.4)	204 104 (20.7)	1 433 244 (53.2)	2 055 568 (52.4)	16 492 (40.7)	2 253 366 (22.9)	157 580 (39.7)
Born outside USA	1676 (1.1)	809 378 (82.0)	461 175 (17.1)	1 879 845 (48.0)	26 728 (66.0)	596 159 (6.1)	36 986 (9.3)
Clinical characteristics							
Parity							
Primiparous*	38 906 (25.9)	366 148 (37.1)	756 993 (28.1)	1 147 275 (29.3)	10 299 (25.4)	3 183 618 (32.4)	130 980 (33.0)
Multiparous with 1 previous birth	34 592 (23.0)	330 770 (33.5)	659 469 (24.5)	1 041 038 (26.6)	8893 (22.0)	2 855 918 (29.1)	103 980 (26.2)
Multiparous with ≥ 2 previous births	76 814 (51.1)	290 105 (29.4)	1 276 425 (47.4)	1 731 154 (44.2)	21 294 (52.6)	3 780 258 (38.5)	161 879 (40.8)
Mode of delivery							
Spontaneous vaginal	105 431 (70.1)	618 813 (62.7)	1 691 646 (62.8)	2 618 161 (66.8)	27 042 (66.8)	6 618 242 (67.4)	271 900 (68.5)
Forceps	559 (0.4)	8276 (0.8)	10 945 (0.4)	16 422 (0.4)	293 (0.7)	60 339 (0.6)	2192 (0.6)
Vacuum	2811 (1.9)	42 128 (4.3)	57 069 (2.1)	78 536 (2.0)	920 (2.3)	259 860 (2.6)	9598 (2.4)
Cesarean with trial of labor	12 086 (8.0)	100 496 (10.2)	283 192 (10.5)	319 652 (8.2)	3569 (8.8)	856 470 (8.7)	37 065 (9.3)
Cesarean without trialof labor	29 425 (19.6)	217 310 (22.0)	650 035 (24.1)	886 696 (22.6)	8662 (21.4)	2 024 883 (20.6)	76 084 (19.2)
Prepregnancy BMI							
Underweight (< 18.5 kg/m^2^)	3156 (2.1)	65 836 (6.7)	80 696 (3.0)	96 938 (2.5)	646 (1.6)	298 773 (3.0)	13 460 (3.4)
Normal (18.5–24.9 kg/m^2^)	45 285 (30.1)	585 947 (59.4)	860 561 (32.0)	1 416 844 (36.1)	9773 (24.1)	4 443 483 (45.3)	156 135 (39.3)
Overweight (25.0–29.9 kg/m^2^)	41 012 (27.3)	235 048 (23.8)	722 929 (26.8)	1 191 455 (30.4)	10 729 (26.5)	2 497 516 (25.4)	102 664 (25.9)
Obese (≥ 30.0 kg/m^2^)	60 859 (40.5)	100 192 (10.2)	1 028 701 (38.2)	1 214 230 (31.0)	19 338 (47.8)	2 580 022 (26.3)	124 580 (31.4)
Assisted reproductive technology	227 (0.2)	20 279 (2.1)	9618 (0.4)	14 359 (0.4)	75 (0.2)	130 213 (1.3)	2878 (0.7)
Prepregnancy diabetes	3916 (2.6)	10 496 (1.1)	35 038 (1.3)	44 476 (1.1)	771 (1.9)	77 200 (0.8)	3966 (1.0)
Prepregnancy hypertension	4341 (2.9)	11 389 (1.2)	113 016 (4.2)	57 388 (1.5)	741 (1.8)	201 163 (2.0)	9669 (2.4)
Gestational DM	15 860 (10.6)	134 455 (13.6)	152 450 (5.7)	296 817 (7.6)	3864 (9.5)	611 331 (6.2)	26 132 (6.6)
Pre‐eclampsia/eclampsia	14 465 (9.6)	45 087 (4.6)	248 233 (9.2)	251 017 (6.4)	3404 (8.4)	791 736 (8.1)	32 291 (8.1)
Induction of labor	45 741 (30.4)	246 306 (25.0)	742 987 (27.6)	1 021 030 (26.1)	8750 (21.6)	3 216 558 (32.8)	121 968 (30.7)
Augmentation of labor	35 174 (23.4)	253 067 (25.6)	554 361 (20.6)	902 017 (23.0)	10 612 (26.2)	2 188 031 (22.3)	96 210 (24.2)
Gestational age at delivery							
Preterm (< 37 weeks)	20 036 (13.3)	88 310 (8.9)	406 533 (15.1)	433 048 (11.0)	6286 (15.5)	847 126 (8.6)	42 406 (10.7)
Term (37–41 weeks)	120 424 (80.1)	855 284 (86.7)	2 153 091 (80.0)	3 259 614 (83.2)	31 027 (76.6)	8 436 475 (85.9)	330 759 (83.3)
Post‐term (≥ 42 weeks)	9852 (6.6)	43 429 (4.4)	133 263 (4.9)	226 805 (5.8)	3173 (7.8)	536 193 (5.5)	23 674 (6.0)
Birth weight ≥ 4000 g	15 850 (10.5)	41 126 (4.2)	118 039 (4.4)	266 420 (6.8)	3755 (9.3)	950 785 (9.7)	29 487 (7.4)

Data are given as *n* (%). *One birth, which is the index birth. AIAN, American Indian or Alaska Native; BMI, body mass index; DM, diabetes mellitus; GED, general educational development; NHOPI, Native Hawaiian and other Pacific Islander; WIC, Women, Infants and Children.

Among all births, 66.4% were SVD, 0.5% were OVD with forceps and 2.5% were OVD with vacuum. The mode of delivery did not differ substantially across race categories, except for OVD with vacuum, for which Asian individuals had approximately double the rate compared with the other groups. However, when analyzed by parity and obstetric history, differences in mode of delivery were observed between racial and ethnic populations. In primiparous individuals, Asian and NHOPI populations had higher rates of OVD with forceps, while a lower rate was observed among Black individuals (Table S2). Among the multiparous population without a previous CD, a higher rate of OVD with vacuum was noted among Asian individuals, while AIAN individuals had a lower rate (Table S3). Similar trends were noted among multiparous individuals with at least one previous CD (Table S4).

**Figure 2:**
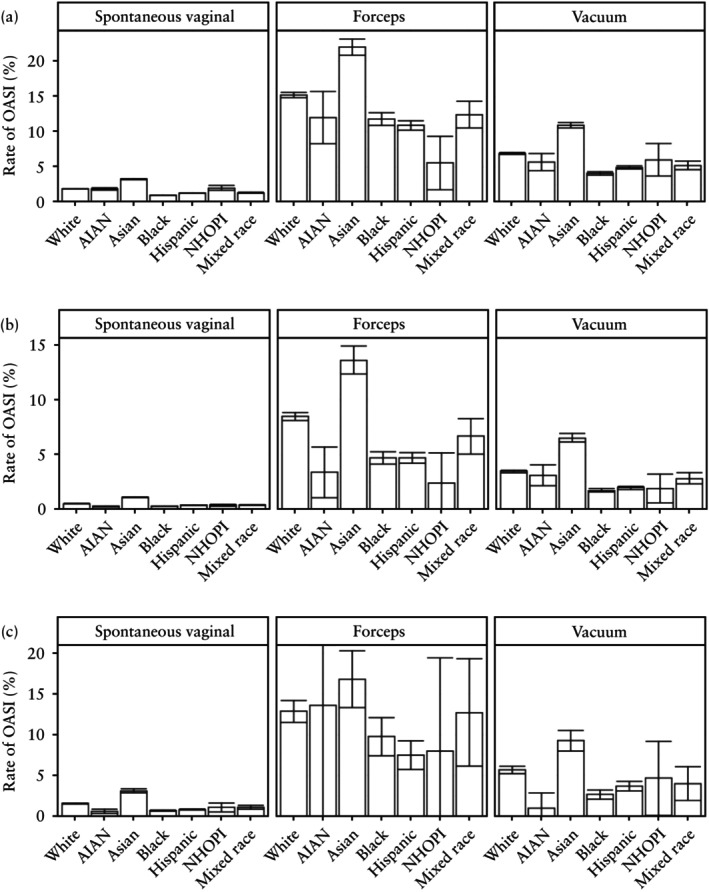
Rate of obstetric anal sphincter injury (OASI) in individuals who had a live singleton birth in the USA between 2016 and 2021, according to race and ethnicity and mode of delivery, among: (a) primiparous individuals, (b) multiparous individuals without previous Cesarean delivery (CD) and (c) multiparous individuals with previous CD. Whiskers represent 95% CIs. AIAN, American Indian or Alaska Native; NHOPI, Native Hawaiian and other Pacific Islander.

In total, 12 501 183 vaginal births were included in our statistical models. Among these, OASI rates varied by parity and obstetric history, with a rate of 2.2% in primiparous individuals, 0.6% in multiparous individuals without a previous CD and 1.6% in multiparous individuals with at least one previous CD. The rate of OASI further varied by mode of delivery within each parity and obstetric‐history stratum. Specifically, among primiparae, those who had OVD with forceps had the highest rate of OASI (14.8%) followed by OVD with vacuum (6.6%) and SVD (1.7%) (Figure 2, Table S5). Among multiparous individuals without a previous CD, rates of OASI were lower than in primiparae (SVD, 0.5%; OVD with forceps, 7.5%; OVD with vacuum, 3.2%), while rates among multiparous individuals with a previous CD were similar to those seen in the primiparous group (SVD, 1.3%; OVD with forceps, 11.8%; OVD with vacuum, 5.1%).

Asian individuals had the highest rate of OASI, irrespective of parity, obstetric history and mode of delivery (Figure 2, Table S5). Among this population, the rates of OASI when undergoing SVD, OVD with forceps and OVD with vacuum were 3.3%, 22.0% and 10.9%, respectively, in primiparous individuals; 1.1%, 13.6% and 6.5%, respectively, in multiparous individuals without a previous CD; and 3.1%, 16.8% and 9.3%, respectively, in multiparous individuals with at least one previous CD. Conversely, in the primiparous group, Black individuals had the lowest rate of OASI of those who had SVD (1.0%) and OVD with vacuum (4.1%), while NHOPI individuals had the lowest rate of OASI of those who had OVD with forceps (5.6%). These trends were consistent in multiparous individuals without a previous CD, while in the multiparous group with a previous CD, the lowest OASI rates were among AIAN (SVD, 0.6%; OVD with vacuum, 1.0%) and Hispanic (OVD with forceps, 7.5%) individuals (Figure 2, Table S5).

**Figure 3:**
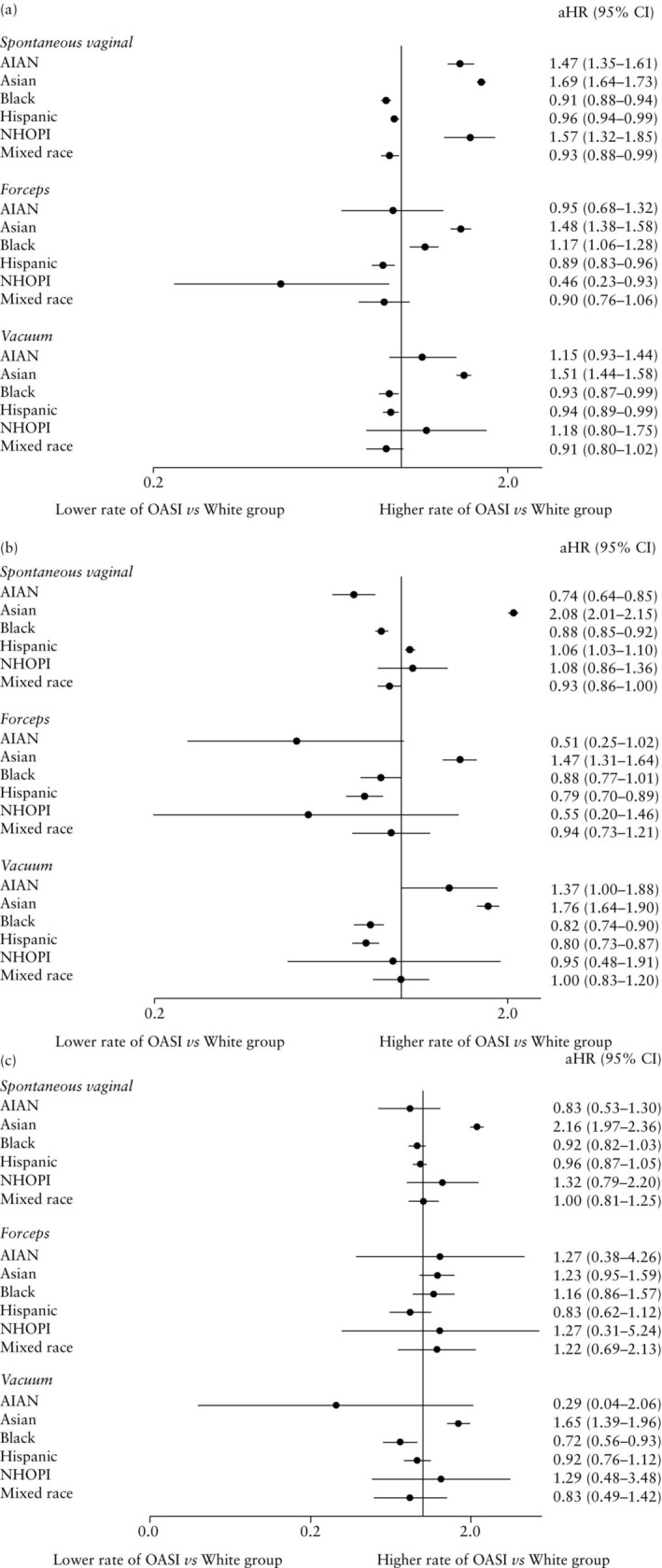
Adjusted hazard ratio (aHR) (95% CI) of obstetric anal sphincter injury (OASI) according to race and ethnicity, compared with White individuals, and according to mode of delivery, in primiparous individuals (a), multiparous individuals without previous Cesarean delivery (CD) (b) and multiparous individuals with previous CD (c), who gave birth in the USA between 2016 and 2021. AIAN, American Indian or Alaska Native; NHOPI, Native Hawaiian and other Pacific Islander.

In primiparous individuals, OASI hazards were 47.0–69.0% higher in those who had SVD for AIAN, Asian and NHOPI *vs* White individuals, while the hazards were 4.0–9.0% lower for Black, Hispanic and mixed‐race individuals (Figure 3a). Similar trends were observed among Asian primiparae who had OVD with forceps or vacuum in the index birth, as well as Black and Hispanic primiparae who had OVD with vacuum (Figure 3a). Conversely, Black individuals had a 17.0% higher hazard of OASI compared with White individuals for those who had OVD with forceps (aHR, 1.17 (95% CI, 1.06–1.28)), while Hispanic primiparae who had OVD with forceps had an 11.0% lower hazard of OASI (aHR, 0.89 (95% CI, 0.83–0.96)). Among primiparae, PAFs revealed that 4.7% of SVD OASI cases, 4.4% of OVD with forceps OASI cases and 5.6% of OVD with vacuum OASI cases could have been avoided if the differences in the experience of racism between Asian and White individuals had been eliminated.

In multiparae without a previous CD, Asian individuals had an increase in OASI hazard of 1.5‐fold to over 2‐fold compared with White individuals (SVD: aHR, 2.08 (95% CI, 2.01–2.15); OVD with forceps: aHR, 1.47 (95% CI, 1.31–1.64)) and Hispanic individuals who had a SVD had a 6.0% higher OASI hazard (aHR, 1.06 (95% CI, 1.03–1.10)) (Figure 3b). This finding differed among Hispanic multiparae who had an OVD with forceps or vacuum, who had an approximately 20% lower hazard of OASI (OVD with forceps: aHR, 0.79 (95% CI, 0.70–0.89); OVD with vacuum: aHR, 0.80 (95% CI, 0.73–0.87)). Similarly, Black individuals who had SVD or OVD with vacuum had a 12.0–18.0% lower hazard of OASI (SVD: aHR, 0.88 (95% CI, 0.85–0.92); OVD with vacuum: aHR, 0.82 (95% CI, 0.74–0.90)) (Figure 3b). Among those who had OVD with forceps, there was a similar hazard for OASI between Black and White individuals. Across the multiparae without a previous CD, PAFs highlighted that 5.7% of SVD OASI cases, 4.1% of OVD with forceps OASI cases and 7.4% of OVD with vacuum OASI cases could have been avoided if the differences in the experience of racism between Asian and White individuals had been eliminated.

Among multiparae with at least one previous CD, Asian individuals who had a SVD had a 2.16 (95% CI, 1.97–2.36) times higher hazard of OASI compared with White individuals, and those who had an OVD with vacuum had a 65.0% higher hazard of OASI (aHR, 1.65 (95% CI, 1.39–1.96)) (Figure 3c). Conversely, Black individuals who had an OVD with vacuum had an almost 30.0% lower OASI hazard when compared with White individuals (aHR, 0.72 (95% CI, 0.56–0.93)) (Figure 3c). Relative estimates of OASI were constrained by a limited number of births in some racial and ethnic groups among multiparae with a previous CD who had OVD with forceps in the index birth. PAFs among multiparae with a previous CD revealed that 4.5% of SVD OASI cases and 7.4% of OVD with vacuum OASI cases could have been avoided if the differences in the experience of racism between Asian and White individuals had been eliminated.

Restricting multiparae with at least one previous CD to those with only one prior birth resulted in a population of 114 540 individuals (Figure S1, Table S6). The primary differences between this analysis and our main models were an attenuation of the relatively higher hazard of OASI in Asian individuals who had a SVD compared with White individuals (rate of OASI, 4.6% *vs* 2.8%; aHR, 1.70 (95% CI, 1.52–1.91)) and an increased difference in the relative hazard among Hispanic individuals who had a SVD compared with White individuals (rate of OASI, 1.6% *v*s 2.8%; aHR, 0.80 (95% CI, 0.71–0.91)) (Table S7).

Analyses with multiple imputation for missing data yielded comparable relative rates of OASI among primiparous and multiparous without a previous CD groups. In multiparous individuals with a previous CD who had a forceps delivery, the hazard of OASI was higher among all racial and ethnic minority groups compared with White individuals, except for Hispanic individuals, who had a 26.0% lower hazard of OASI (rate of OASI, 7.5% *vs* 12.9%; aHR, 0.74 (95% CI, 0.68–0.81)) (Table S8). The relative hazards of OASI among multiparous individuals with a previous CD who had an OVD with vacuum were comparable with our main models for all racial and ethnic groups. When corrected for outcome misclassification and unmeasured confounding, our estimates varied in attenuating and widening the association of race and ethnicity with OASI (Figure S2). Notably, disparities in OASI rates were attenuated among Asian individuals and increased among Black individuals.

**Figure 4:**
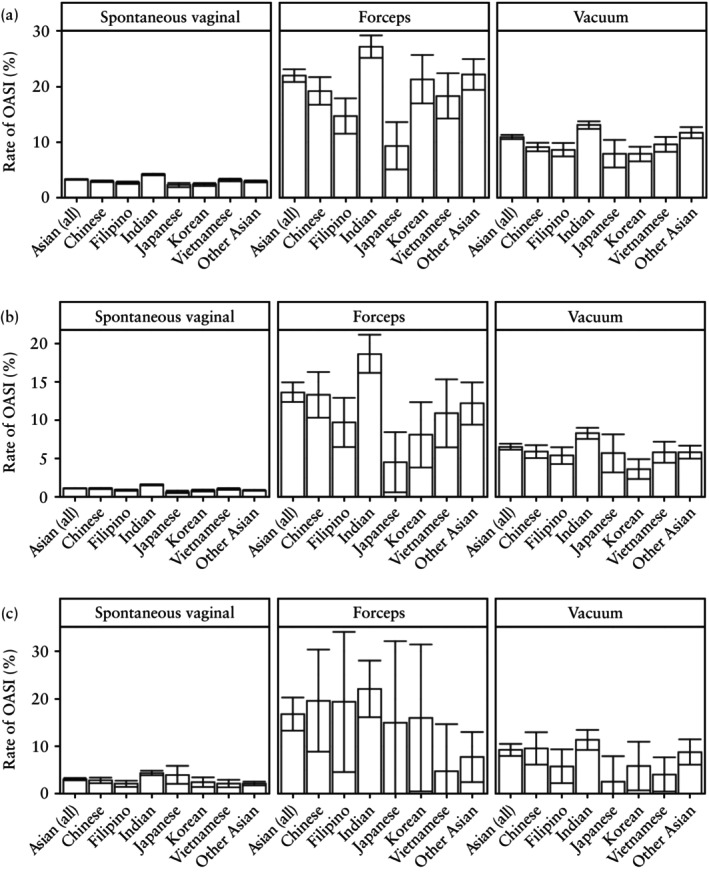
Rate of obstetric anal sphincter injury (OASI) in Asian ethnic subgroups who delivered in the USA between 2016 and 2021, according to mode of delivery, in: (a) primiparous individuals, (b) multiparous individuals without previous Cesarean delivery (CD) and (c) multiparous individuals with previous CD. Whiskers represent 95% CIs.

Considering disaggregated Asian racial subgroups, we explored which specific groups might be driving the high OASI rate among Asian individuals. In the primiparous population, individuals with origins and/or ancestry from India had the highest OASI rates across delivery modes (SVD, 4.2%; OVD with forceps, 27.2%; OVD with vacuum, 13.1%) and the lowest OASI rate was among individuals with origins and/or ancestry from Japan (SVD, 2.3%; OVD with forceps, 9.3%; OVD with vacuum, 7.9%) (Figure 4a, Table S9). Compared with the aggregate Asian group, the rate of OASI was higher among Indian and ‘other’ Asian individuals (Figure 4a). Similar trends were noted in the multiparous population without a previous CD, while comparisons in the multiparous population with a previous CD were constrained by the small numbers of births in some groups (Figure 4b,c). Overall, Indian individuals had OASI hazards that were 62.0–173.0% higher than those of White individuals (Table S10).

**Figure 5:**
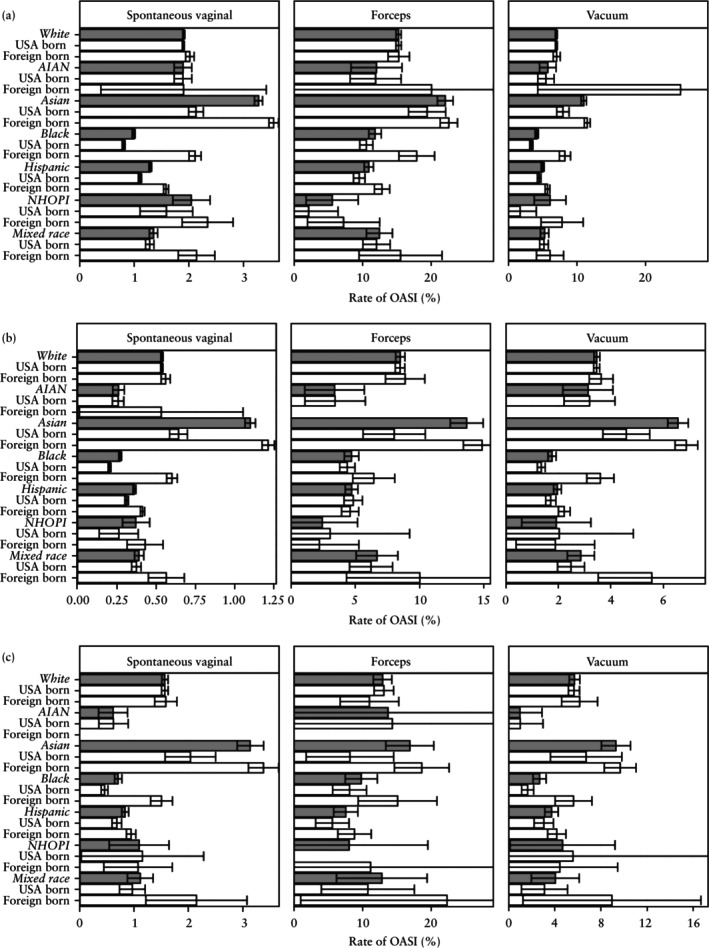
Rate of obstetric anal sphincter injury (OASI) among foreign‐born *vs* USA‐born individuals who delivered in the USA between 2016 and 2021, according to race and ethnicity and mode of delivery, in: (a) primiparous individuals, (b) multiparous individuals without previous Cesarean delivery (CD) and (c) multiparous individuals with previous CD. Note that the limited number of births in some groups resulted in large 95% CIs; these findings should be interpreted with caution. Whiskers represent 95% CIs. AIAN, American Indian or Alaska Native; NHOPI, Native Hawaiian and other Pacific Islander.

USA‐born and foreign‐born White individuals had similar OASI rates across parity, obstetric‐history and mode‐of‐delivery groups. Conversely, in many of the study strata, higher OASI rates were noted among foreign‐born *vs* USA‐born individuals among Asian, Black, Hispanic and mixed‐race populations (Figure 5). The limited number of observations in AIAN and NHOPI populations hindered comparisons of OASI rate by place of birth in these groups.

## DISCUSSION

This study highlights differences in rates of OASI by race and ethnicity that vary in magnitude and direction based on maternal and obstetric characteristics. The key findings include the consistently high rates of OASI among Asian individuals across subgroups, and the more pronounced disparities among Asian *vs* White multiparae compared with among Asian *vs* White primiparae, in some strata. For instance, among those who had a SVD, Asian primiparae had a 69% higher OASI hazard, while Asian multiparae with a previous CD had a 116% higher OASI hazard, compared with the respective White groups. We further demonstrated that the high OASI rates among Asian individuals were largely driven by individuals with origins and/or ancestry from India and those who were born outside the USA. These findings indicate that valuable information is lost with the use of aggregate race and ethnicity groupings, impacting policy and resource allocation for populations most at risk for OASI. Future research is needed to understand the reasons behind the disproportionately higher OASI rates among Asian and, more specifically, Indian individuals.

Black and Hispanic individuals had lower OASI rates across many of the parity, obstetric‐history and mode‐of‐delivery groups. While these findings are consistent with those of the previous literature^4^, ^5^, ^6^, they indicate that disparities in OASI rates cannot be solely attributed to structural racism. Instead, factors such as language and immigration status may interact with racism to shape an individual's risk of OASI. Research in health equity has emphasized the importance of studying race and ethnicity alongside interrelated socioeconomic factors to overcome the limitations of race as a surrogate measure and to account for various levels of marginalization on maternal health^27^, ^28^. Our results show a higher rate of OASI among foreign‐born Black individuals *vs* their USA‐born counterparts, which was also higher than that in White individuals in some strata. This finding highlights the heterogeneity of the Black racial and ethnic group, as collected from files regarding place of birth. Studies conducted in other high‐income countries have found individuals from Sub‐Saharan Africa to have a 1.60–3.82 times higher risk of OASI compared with Norwegian‐ and Swedish‐born individuals, respectively^29^, ^30^. The disaggregation of the Black racial and ethnic category in the USA and adoption of an intersectional research approach will assist in advancing our understanding of Black–White disparities in maternal perineal health.

Several studies have investigated potential causal mechanisms of Asian–White disparities in OASI. For instance, research has analyzed perineal length and found no differences between races, no association between perineal length and perineal lacerations, and among Chinese individuals, no effect of maternal–fetal size disposition or BMI on OASI risk^7^. According to Howell's conceptual model on health disparities^28^, care received across the preconception, antenatal, delivery and postpartum periods can have an impact on maternal health. A Canadian study examining prenatal counseling practices found that 65% of patients did not receive information regarding the risks of OVD^31^, which is a strong determinant for OASI^4^. Additionally, a recent systematic review highlighted that language barriers among the Asian population in Australia can cause confusion between the words ‘breathe’ and ‘push’ during delivery and that there is limited access to language interpretation services in the USA for pregnant Korean individuals^7^. A Norway‐based study also highlighted the facilitating role of a spouse/partner during labor and delivery, as well as migration‐related factors, with the risk of OASI among South Asian individuals attenuating if they had a Norwegian‐born partner and if they had longer residence in the country^30^. It is important to emphasize that race is a social construct, and thus it is not race and ethnicity that confer a higher OASI risk but racism^7^. The persistently high OASI rate among Asian and, more specifically, Indian individuals in the USA calls for an investigation of healthcare services across the continuum of care for this population, their integration, cultural sensitivity and effectiveness in providing culturally safe care. These efforts will be instrumental in addressing maternal‐health inequalities at a system, provider and patient level.

Our study is constrained by the sensitivity of the OASI indicator, resulting in an under‐representation of OASI cases^22^ and a lack of information on some confounders, such as episiotomy, previous OASI, length of residence in the USA and hospital‐level factors. We mitigated these limitations through a probabilistic bias analysis, which shifted our estimates of OASI both towards and away from the null value, depending on race and ethnicity. Additionally, relative estimates in the multiparous population with a previous CD may have been biased by the inability to account for the number of vaginal deliveries between a previous CD and the index birth. The sensitivity analysis restricted to multiparae with one prior birth found the OASI hazard to be mostly unchanged. However, we did observe an attenuation in OASI hazard among Asian *vs* White individuals who had a SVD and a decreased hazard among Hispanic individuals. Lastly, this study used population‐based data specific to the USA, which limits its generalizability. However, several countries, e.g. Sweden, Norway, Italy, Australia and New Zealand, have reported similar patterns of racial disparities in OASI^30^, ^32^, ^33^, ^34^.

Strengths of our study include that it is an analysis of a large, nationally representative population, facilitating the stratification of individuals into groups with a similar risk for OASI. The use of stratification yielded easily interpretable results that could be compared across strata, providing a nuanced understanding of how OASI risk is influenced by parity, obstetric history and mode of delivery, that would otherwise be masked by adjusting for these determinants. Moreover, detailed information on self‐reported race and maternal and obstetric characteristics, as well as consistent data collection over the study years, allowed for a robust analysis.

In conclusion, racial disparities in rates of OASI are apparent across maternal and obstetric characteristics. There is an association of race and ethnicity with OASI such that Asian individuals have an increased rate of OASI compared with White individuals. These disparities call for investigations into other clinical and non‐clinical factors that may be responsible for the association of race and ethnicity with OASI. Such efforts will be instrumental in designing healthcare services for populations most at risk for OASI that are culturally sensitive, trauma‐informed and implemented across the continuum of care. By doing so, we could address plausible root causes for health inequities in OASI.

## Supporting information


**Figure S1** Flowchart showing derivation of study strata by parity and obstetric history.
**Figure S2** Bias analysis correcting for outcome misclassification and unmeasured confounding for obstetric anal sphincter injury by race and ethnicity and mode of delivery, among primiparous and multiparous individuals (sensitivity analysis) who delivered in the USA between 2016 and 2021.
**Tables S1–S4** Demographic and clinical characteristics of 3 855 160 individuals with missing information (Table S1), 5 634 219 primiparous individuals (Table S2), 9 610 026 multiparous individuals without previous Cesarean delivery (Table S3) and 2 762 563 multiparous individuals with previous Cesarean delivery (Table S4), who delivered in the USA between 2016 and 2021
**Table S5** Rate of obstetric anal sphincter injury by race and ethnicity in primiparous and multiparous individuals who delivered in the USA between 2016 and 2021
**Table S6** Rate of obstetric anal sphincter injury by race and ethnicity in 114 540 multiparous individuals with at least one previous Cesarean delivery and one prior birth (sensitivity analysis) who delivered in the USA between 2016 and 2021
**Table S7** Crude and adjusted hazard ratios (HRs) and 95% CIs for obstetric anal sphincter injury by mode of delivery, among 114 540 multiparous individuals with at least one previous Cesarean delivery and one prior birth (sensitivity analysis) who delivered in the USA between 2016 and 2021
**Table S8** Crude and adjusted hazard ratios (HRs) and 95% CIs for obstetric anal sphincter injury by race and ethnicity and mode of delivery, among primiparous and multiparous individuals who delivered in the USA between 2016 and 2021, using multiple imputation for missing data (sensitivity analysis)
**Table S9** Rates of obstetric anal sphincter injury by Asian race subcategories among spontaneous vaginal, forceps and vacuum delivery, stratified by parity and obstetric history
**Table S10** Crude and adjusted hazard ratios (HRs) and 95% CIs for obstetric anal sphincter injury by Asian race subcategories and mode of delivery, stratified by parity and obstetric history (sensitivity analysis)

## Data Availability

The data that support the findings of this study are openly available in Vital Statistics Online Data Portal at https://www.cdc.gov/nchs/data_access/vitalstatsonline.htm.
