# Supplementary material for: Racial and ethnic disparities in obstetric anal sphincter injury: cross‐sectional study in the USA
Source: Ultrasound Obstet Gynecol. 2025 May 9;65(6):778–89. doi: 10.1002/uog.29231 (PMC12127723; doi:10.1002/uog.29231)
Supplement: Supplementary file 1 — Figure S1 Flowchart showing derivation of study strata by parity and obstetric history. Figure S2 Bias analysis correcting for outcome misclassification and unmeasured confounding for obstetric anal sphincter injury by race and ethnicity and mode of delivery, among primiparous and multiparous individuals (sensitivity analysis) who delivered in the USA between 2016 and 2021. Tables S1–S4 Demographic and clinical characteristics of 3 855 160 individuals with missing information (Table S1), 5 634 219 primiparous individuals (Table S2), 9 610 026 multiparous individuals without previous Cesarean delivery (Table S3) and 2 762 563 multiparous individuals with previous Cesarean delivery (Table S4), who delivered in the USA between 2016 and 2021 Table S5 Rate of obstetric anal sphincter injury by race and ethnicity in primiparous and multiparous individuals who delivered in the USA between 2016 and 2021 Table S6 Rate of obstetric anal sphincter injury by race and ethnicity in 114 540 multiparous individuals with at least one previous Cesarean delivery and one prior birth (sensitivity analysis) who delivered in the USA between 2016 and 2021 Table S7 Crude and adjusted hazard ratios (HRs) and 95% CIs for obstetric anal sphincter injury by mode of delivery, among 114 540 multiparous individuals with at least one previous Cesarean delivery and one prior birth (sensitivity analysis) who delivered in the USA between 2016 and 2021 Table S8 Crude and adjusted hazard ratios (HRs) and 95% CIs for obstetric anal sphincter injury by race and ethnicity and mode of delivery, among primiparous and multiparous individuals who delivered in the USA between 2016 and 2021, using multiple imputation for missing data (sensitivity analysis) Table S9 Rates of obstetric anal sphincter injury by Asian race subcategories among spontaneous vaginal, forceps and vacuum delivery, stratified by parity and obstetric history Table S10 Crude and adjusted hazard ratios (HRs) and 95% CIs for obstetric anal sph [file UOG-65-778-s001.docx]

**Table of contents**

[Appendix S1. Flowchart showing derivation of study strata by parity and obstetric history 2](#_Toc192068008)

[Appendix S2. Demographic and clinical characteristics of individuals with missing information, United States, 2016-2021 (N=3,855,160)^*^ 3](#_Toc192068009)

[Appendix S3. Demographic and clinical characteristics of primiparous individuals, United States, 2016-2021 (N=5,634,219)^*^ 6](#_Toc192068010)

[Appendix S4. Demographic and clinical characteristics of multiparous individuals without a previous cesarean delivery, United States, 2016-2021 (N=9,610,026)^*^ 8](#_Toc192068011)

[Appendix S5. Demographic and clinical characteristics of multiparous individuals with a previous cesarean delivery, United States, 2016-2021 (N=2,762,563)^*^ 10](#_Toc192068012)

[Appendix S6. Rate of obstetric anal sphincter injury (%) by race and ethnicity in primiparous and multiparous individuals, United States 2016-2021 12](#_Toc192068013)

[Appendix S7. Rate of obstetric anal sphincter injury (%) by race and ethnicity in multiparous individuals with at least one previous cesarean delivery and one prior birth (sensitivity analysis), United States 2016-2021 (N=114,540) 13](#_Toc192068014)

[Appendix S8. Crude and adjusted hazard ratios (HRs) and 95% confidence intervals (CIs) for obstetric anal sphincter injury by mode of delivery, among multiparous individuals with at least one previous cesarean delivery and one prior birth (sensitivity analysis), United States, 2016-2021 (N=114,540)^*^ 14](#_Toc192068015)

[Appendix S9. Crude and adjusted hazard ratios (HRs) and 95% confidence intervals (CIs) for obstetric anal sphincter injury by race and ethnicity and mode of delivery, among primiparous and multiparous individuals using multiple imputation for missing data (sensitivity analysis), United States, 2016-2021^*^ 15](#_Toc192068016)

[Appendix S10. Bias analysis correcting for outcome misclassification and unmeasured confounding for obstetric anal sphincter injury by race and ethnicity and mode of delivery, among primiparous and multiparous individuals (sensitivity analysis), United States, 2016-2021 16](#_Toc192068017)

[Appendix S11. Rates of obstetric anal sphincter injury by Asian race subcategories among spontaneous vaginal, forceps, and vacuum delivery, stratified by parity and obstetric history, United States, 2016-2021 17](#_Toc192068018)

[Appendix S12. Crude and adjusted hazard ratios (HRs) and 95% confidence intervals (CIs) for obstetric anal sphincter injury by Asian race subcategories and mode of delivery, stratified by parity and obstetric history (sensitivity analysis), United States, 2016-2021^*^ 18](#_Toc192068019)

# **Appendix S1. Flowchart showing derivation of study strata by parity and obstetric history**


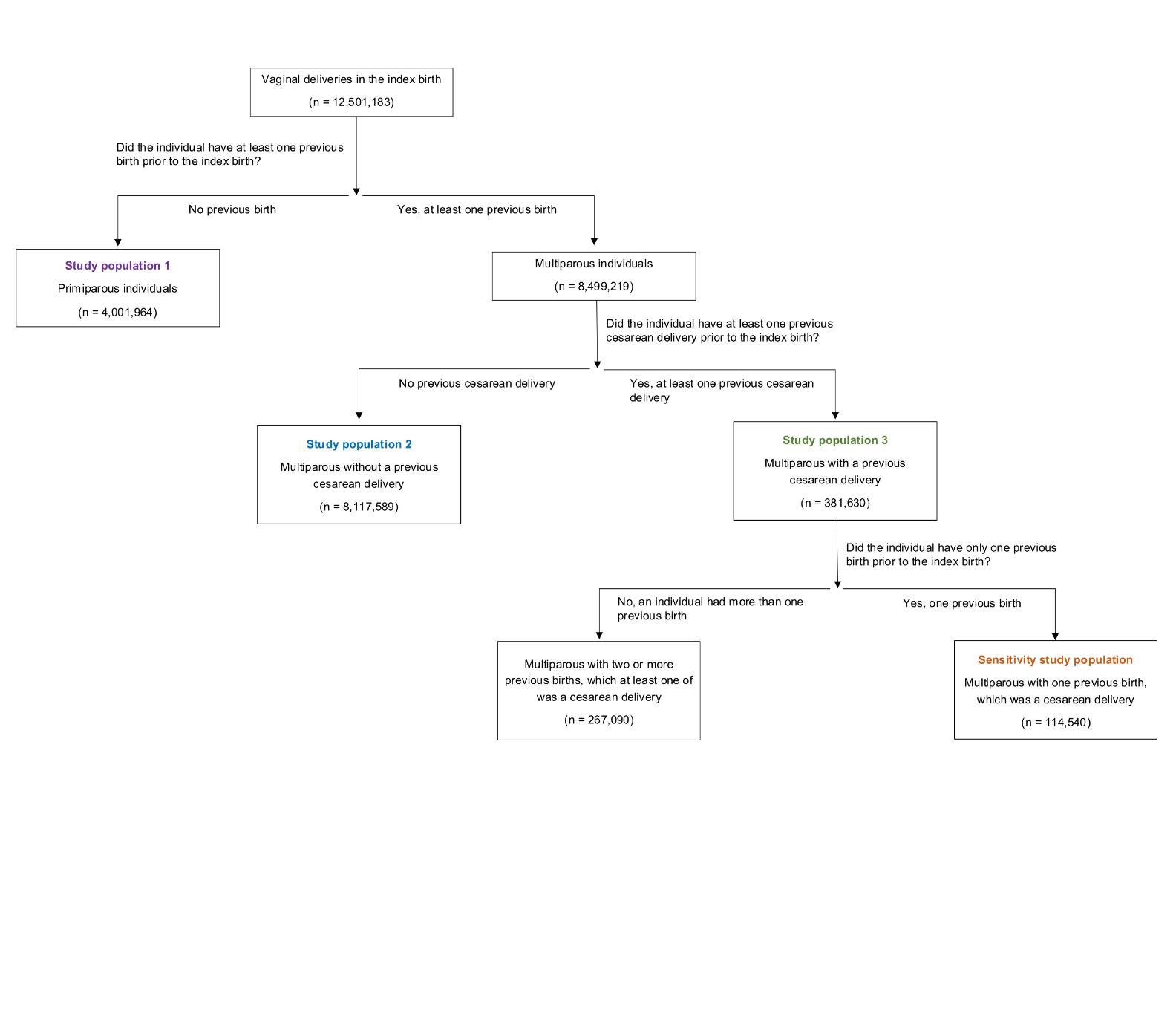


# **Appendix S2. Demographic and clinical characteristics of individuals with missing information, United States, 2016-2021 (N=3,855,160)^*^**

|  | AIAN  (n=16,972) | Asian  (n=389,592) | Black  (n=437,177) | Hispanic  (n= 1,288,216) | NHOPI (n=15,197) | White  (n=1,420,624) | Multiple race (n=88,468) | Missing race (n=198,914) |
| --- | --- | --- | --- | --- | --- | --- | --- | --- |
| Patient demographics | |  |  |  |  |  |  |  |
| Age (years) |  |  |  |  |  |  |  |  |
| ≤ 19 | 1,351 (8.0) | 1,800 (0.5) | 30,936 (7.1) | 81,279 (6.3) | 666 (4.4) | 44,054 (3.1) | 5,429 (6.1) | 6,400 (3.2) |
| 20-24 | 4,231 (24.9) | 18,153 (4.7) | 108,844 (24.9) | 284,184 (22.1) | 3,489 (23.0) | 216,497 (15.2) | 19,053 (21.5) | 26,946 (13.5) |
| 25-29 | 5,093 (30.0) | 80,386 (20.6) | 129,287 (29.6) | 372,802 (28.9) | 4,504 (29.6) | 386,036 (27.2) | 23,989 (27.1) | 47,950 (24.1) |
| 30-34 | 3,825 (22.5) | 157,412 (40.4) | 99,497 (22.8) | 318,486 (24.7) | 3,861 (25.4) | 462,266 (32.5) | 23,463 (26.5) | 62,498 (31.4) |
| 35-40 | 2,016 (11.9) | 105,872 (27.2) | 54,382 (12.4) | 181,957 (14.1) | 2,124 (14.0) | 255,351 (18.0) | 13,413 (15.2) | 41,569 (20.9) |
| > 40 | 456 (2.7) | 25,969 (6.7) | 14,231 (3.3) | 49,508 (3.8) | 553 (3.6) | 56,420 (4.0) | 3,121 (3.5) | 13,551 (6.8) |
| Marital Status | |  |  |  |  |  |  |  |
| Unmarried | 2,925 (17.2) | 67,518 (17.3) | 89,689 (20.5) | 131,452 (10.2) | 2,876 (18.9) | 569,879 (40.1) | 15,404 (17.4) | 75,455 (37.9) |
| Married | 7,373 (43.4) | 9,147 (2.3) | 241,781 (55.3) | 156,300 (12.1) | 4,048 (26.6) | 268,871 (18.9) | 20,759 (23.5) | 46,347 (23.3) |
| Unknown | 6,674 (39.3) | 312,927 (80.3) | 105,707 (24.2) | 1,000,464 (77.7) | 8,273 (54.4) | 581,874 (41.0) | 52,305 (59.1) | 77,112 (38.8) |
| Education |  |  |  |  |  |  |  |  |
| ≤ 8^th^ grade | 229 (1.3) | 4,293 (1.1) | 6,058 (1.4) | 108,501 (8.4) | 350 (2.3) | 20,295 (1.4) | 642 (0.7) | 2,685 (1.3) |
| High school, N.D | 3,169 (18.7) | 7,623 (2.0) | 49,699 (11.4) | 188,400 (14.6) | 2,036 (13.4) | 73,881 (5.2) | 6,856 (7.7) | 8,451 (4.2) |
| High school, or GED | 6,206 (36.6) | 39,805 (10.2) | 146,263 (33.5) | 408,164 (31.7) | 5,670 (37.3) | 283,838 (20.0) | 21,470 (24.3) | 22,348 (11.2) |
| College, N.D | 3,675 (21.7) | 40,154 (10.3) | 109,490 (25.0) | 264,421 (20.5) | 3,370 (22.2) | 267,648 (18.8) | 22,663 (25.6) | 15,338 (7.7) |
| Associate | 1,000 (5.9) | 24,501 (6.3) | 30,649 (7.0) | 76,859 (6.0) | 1,056 (6.9) | 116,116 (8.2) | 7,088 (8.0) | 6,135 (3.1) |
| Bachelor’s | 1,038 (6.1) | 141,178 (36.2) | 44,051 (10.1) | 122,779 (9.5) | 1,235 (8.1) | 372,678 (26.2) | 17,106 (19.3) | 18,220 (9.2) |
| Masters | 311 (1.8) | 82,615 (21.2) | 19,707 (4.5) | 38,411 (3.0) | 307 (2.0) | 170,023 (12.0) | 6,909 (7.8) | 9,323 (4.7) |
| Doctorate | 95 (0.6) | 28,646 (7.4) | 4,249 (1.0) | 9,061 (0.7) | 83 (0.5) | 55,650 (3.9) | 2,631 (3.0) | 3,236 (1.6) |
| Unknown | 1,249 (7.4) | 20,777 (5.3) | 27,011 (6.2) | 71,620 (5.6) | 1,090 (7.2) | 60,495 (4.3) | 3,103 (3.5) | 113,178 (56.9) |
| Payer |  |  |  |  |  |  |  |  |
| Medicaid | 9,639 (56.8) | 75,384 (19.3) | 268,232 (61.4) | 753,601 (58.5) | 7,558 (49.7) | 407,906 (28.7) | 35,178 (39.8) | 67,405 (33.9) |
| Private insurance | 3,505 (20.7) | 259,468 (66.6) | 121,548 (27.8) | 410,077 (31.8) | 4,895 (32.2) | 825,939 (58.1) | 42,948 (48.5) | 108,734 (54.7) |
| Self-pay | 419 (2.5) | 35,256 (9.0) | 10,934 (2.5) | 39,973 (3.1) | 870 (5.7) | 49,997 (3.5) | 2,020 (2.3) | 7,942 (4.0) |
| Other | 1,651 (9.7) | 13,594 (3.5) | 17,061 (3.9) | 54,655 (4.2) | 964 (6.3) | 60,240 (4.2) | 4,918 (5.6) | 7,339 (3.7) |
| Unknown | 1,758 (10.4) | 5,890 (1.5) | 19,402 (4.4) | 29,910 (2.3) | 910 (6.0) | 76,542 (5.4) | 3,404 (3.8) | 7,494 (3.8) |
| WIC |  |  |  |  |  |  |  |  |
| No | 7,728 (45.5) | 304,939 (78.3) | 188,068 (43.0) | 515,217 (40.0) | 8,639 (56.8) | 1,015,528 (71.5) | 54,076 (61.1) | 129,940 (65.3) |
| Yes | 6,696 (39.5) | 64,147 (16.5) | 208,943 (47.8) | 723,233 (56.1) | 4,900 (32.2) | 300,459 (21.1) | 27,306 (30.9) | 45,711 (23.0) |
| Unknown | 2,548 (15.0) | 20,506 (5.3) | 40,166 (9.2) | 49,766 (3.9) | 1,658 (10.9) | 104,637 (7.4) | 7,086 (8.0) | 23,263 (11.7) |
| Nativity |  |  |  |  |  |  |  |  |
| US born | 16,408 (96.7) | 93,193 (23.9) | 369,867 (84.6) | 731,051 (56.7) | 6,172 (40.6) | 1,257,435 (88.5) | 79,529 (89.9) | 140,765 (70.8) |
| Foreign born | 335 (2.0) | 291,977 (74.9) | 56,252 (12.9) | 550,459 (42.7) | 8,193 (53.9) | 149,125 (10.5) | 7,927 (9.0) | 52,562 (26.4) |
| Unknown | 229 (1.3) | 4,422 (1.1) | 11,058 (2.5) | 6,706 (0.5) | 832 (5.5) | 14,064 (1.0) | 1,012 (1.1) | 5,587 (2.8) |
| Clinical characteristics | |  |  |  |  |  |  |  |
| Parity | |  |  |  |  |  |  |  |
| Primiparous | 3,952 (23.3) | 155,942 (40.0) | 122,379 (28.0) | 384,275 (29.8) | 4,101 (27.0) | 476,685 (33.6) | 31,603 (35.7) | 64,227 (32.3) |
| Multiparous with 1 previous birth | 3,573 (21.1) | 130,564 (33.5) | 103,699 (23.7) | 341,885 (26.5) | 3,389 (22.3) | 403,986 (28.4) | 23,221 (26.2) | 52,300 (26.3) |
| Multiparous with ≥2 previous births | 8,682 (51.2) | 99,318 (25.5) | 196,153 (44.9) | 546,347 (42.4) | 7,242 (47.7) | 495,793 (34.9) | 31,829 (36.0) | 75,598 (38.0) |
| Missing | 765 (4.5) | 3,768 (1.0) | 14,946 (3.4) | 15,709 (1.2) | 465 (3.1) | 44,160 (3.1) | 1,815 (2.1) | 6,789 (3.4) |
| Mode of delivery |  |  |  |  |  |  |  |  |
| SVD | 11,804 (69.5) | 248,723 (63.8) | 276,499 (63.2) | 874,621 (67.9) | 10,128 (66.6) | 952,492 (67.0) | 60,258 (68.1) | 131,391 (66.1) |
| Forceps | 62 (0.4) | 2,086 (0.5) | 1,535 (0.4) | 3,068 (0.2) | 79 (0.5) | 6,627 (0.5) | 355 (0.4) | 1,133 (0.6) |
| Vacuum | 364 (2.1) | 20,263 (5.2) | 10,303 (2.4) | 31,631 (2.5) | 350 (2.3) | 41,328 (2.9) | 2,519 (2.8) | 6,019 (3.0) |
| Cesarean delivery with TOL | 911 (5.4) | 23,451 (6.0) | 36,911 (8.4) | 63,255 (4.9) | 1,088 (7.2) | 98,489 (6.9) | 6,067 (6.9) | 12,926 (6.5) |
| Cesarean delivery without TOL | 3,774 (22.2) | 94,596 (24.3) | 110,533 (25.3) | 313,528 (24.3) | 3,530 (23.2) | 316,686 (22.3) | 19,069 (21.6) | 43,949 (22.1) |
| Unknown | 57 (0.3) | 473 (0.1) | 1,396 (0.3) | 2,113 (0.2) | 22 (0.1) | 5,002 (0.4) | 200 (0.2) | 3,496 (1.8) |
| Pre-pregnancy BMI (kg/m^2^) | |  |  |  |  |  |  |  |
| Underweight (<18.5) | 278 (1.6) | 24,421 (6.3) | 10,678 (2.4) | 22,904 (1.8) | 185 (1.2) | 42,643 (3.0) | 2688 (3.0) | 6349 (3.2) |
| Normal weight (18.5-24.9) | 4,045 (23.8) | 220,010 (56.5) | 107,590 (24.6) | 375,356 (29.1) | 2,713 (17.9) | 589,378 (41.5) | 33,297 (37.6) | 83,198 (41.8) |
| Overweight (25.0-29.9) | 3,406 (20.1) | 79,808 (20.5) | 90,280 (20.7) | 360,251 (28.0) | 3,028 (19.9) | 303,620 (21.4) | 20,137 (22.8) | 47,624 (23.9) |
| Obese (≥30.0) | 4,868 (28.7) | 34,361 (8.8) | 132,558 (30.3) | 392,462 (30.5) | 5,961 (39.2) | 284,831 (20.0) | 22,169 (25.1) | 42,308 (21.3) |
| Unknown | 4,375 (25.8) | 30,992 (8.0) | 96,071 (22.0) | 137,243 (10.7) | 3,310 (21.8) | 200,152 (14.1) | 10,177 (11.5) | 19,435 (9.8) |
| Assisted reproductive technology | |  |  |  |  |  |  |  |
| No | 15,557 (91.7) | 364,927 (93.7) | 276,255 (63.2) | 1,215,887 (94.4) | 14,483 (95.3) | 985,089 (69.3) | 73,473 (83.1) | 187,506 (94.3) |
| Yes | 29 (0.2) | 8,451 (2.2) | 1,027 (0.2) | 3,889 (0.3) | 49 (0.3) | 15,990 (1.1) | 764 (0.9) | 6,413 (3.2) |
| Unknown | 1,386 (8.2) | 16,214 (4.2) | 159,895 (36.6) | 68,440 (5.3) | 665 (4.4) | 419,545 (29.5) | 14,231 (16.1) | 4,995 (2.5) |
| Pre-pregnancy diabetes | |  |  |  |  |  |  |  |
| No | 16,447 (96.9) | 385,491 (98.9) | 429,015 (98.1) | 1,272,858 (98.8) | 14,874 (97.9) | 1,402,753 (98.7) | 87,431 (98.8) | 194,126 (97.6) |
| Yes | 231 (1.4) | 3,640 (0.9) | 5,743 (1.3) | 12,814 (1.0) | 287 (1.9) | 10,445 (0.7) | 687 (0.8) | 1,519 (0.8) |
| Unknown | 294 (1.7) | 461 (0.1) | 2,419 (0.6) | 2,544 (0.2) | 36 (0.2) | 7,426 (0.5) | 350 (0.4) | 3,269 (1.6) |
| Pre-pregnancy hypertension | |  |  |  |  |  |  |  |
| No | 16,286 (96.0) | 384,864 (98.8) | 416,188 (95.2) | 1,270,326 (98.6) | 14,858 (97.8) | 1,386,120 (97.6) | 86,269 (97.5) | 192,526 (96.8) |
| Yes | 392 (2.3) | 4,267 (1.1) | 18,570 (4.2) | 15,346 (1.2) | 303 (2.0) | 27,078 (1.9) | 1,849 (2.1) | 3,119 (1.6) |
| Unknown | 294 (1.7) | 461 (0.1) | 2,419 (0.6) | 2,544 (0.2) | 36 (0.2) | 7,426 (0.5) | 350 (0.4) | 3,269 (1.6) |
| Gestational diabetes | |  |  |  |  |  |  |  |
| No | 15,307 (90.2) | 343,138 (88.1) | 412,220 (94.3) | 1,190,858 (92.4) | 13,766 (90.6) | 1,332,302 (93.8) | 82,689 (93.5) | 181,631 (91.3) |
| Yes | 1,371 (8.1) | 45,993 (11.8) | 22,538 (5.2) | 94,814 (7.4) | 1,395 (9.2) | 80,896 (5.7) | 5,429 (6.1) | 14,014 (7.0) |
| Unknown | 294 (1.7) | 461 (0.1) | 2,419 (0.6) | 2,544 (0.2) | 36 (0.2) | 7,426 (0.5) | 350 (0.4) | 3,269 (1.6) |
| Preeclampsia/eclampsia | |  |  |  |  |  |  |  |
| No | 15,562 (91.7) | 372,352 (95.6) | 401,252 (91.8) | 1,216,168 (94.4) | 14,079 (92.6) | 1,325,893 (93.3) | 82,133 (92.8) | 185,624 (93.3) |
| Yes | 1,410 (8.3) | 17,240 (4.4) | 35,925 (8.2) | 72,048 (5.6) | 1,118 (7.4) | 94,731 (6.7) | 6,335 (7.2) | 13,290 (6.7) |
| Induction of labor | |  |  |  |  |  |  |  |
| No | 13,058 (76.9) | 313,660 (80.5) | 329,882 (75.5) | 1,043,580 (81.0) | 12,255 (80.6) | 1,028,308 (72.4) | 66,679 (75.4) | 153,159 (77.0) |
| Yes | 3,744 (22.1) | 75,314 (19.3) | 105,745 (24.2) | 242,578 (18.8) | 2,900 (19.1) | 384,368 (27.1) | 21,464 (24.3) | 43,740 (22.0) |
| Unknown | 170 (1.0) | 618 (0.2) | 1,550 (0.4) | 2,058 (0.2) | 42 (0.3) | 7,948 (0.6) | 325 (0.4) | 2,015 (1.0) |
| Augmentation of labor | |  |  |  |  |  |  |  |
| No | 13,462 (79.3) | 300,411 (77.1) | 354,241 (81.0) | 1,045,473 (81.2) | 11,891 (78.2) | 1,143,732 (80.5) | 70,352 (79.5) | 159,902 (80.4) |
| Yes | 3,341 (19.7) | 88,580 (22.7) | 81,496 (18.6) | 240,735 (18.7) | 3,264 (21.5) | 271,361 (19.1) | 17,838 (20.2) | 37,017 (18.6) |
| Unknown | 169 (1.0) | 601 (0.2) | 1,440 (0.3) | 2,008 (0.2) | 42 (0.3) | 5,531 (0.4) | 278 (0.3) | 1,995 (1.0) |
| Gestational age (weeks) | |  |  |  |  |  |  |  |
| Pre-term (<37) | 2,589 (15.3) | 31,587 (8.1) | 70,883 (16.2) | 128,239 (10.0) | 2,278 (15.0) | 128,771 (9.1) | 9.054 (10.2) | 20,815 (10.5) |
| Term (37-41) | 13,221 (77.9) | 347,633 (89.2) | 346,886 (79.3) | 1,113,645 (86.4) | 12,136 (79.9) | 1,224,529 (86.2) | 75,454 (85.3) | 166,523 (83.7) |
| Post-term (≥42) | 853 (5.0) | 9,976 (2.6) | 17,188 (3.9) | 43,719 (3.4) | 691 (4.5) | 61,042 (4.3) | 3,608 (4.1) | 7,813 (3.9) |
| Unknown | 309 (1.8) | 396 (0.1) | 2,220 (0.5) | 2,613 (0.2) | 92 (0.6) | 6,282 (0.4) | 352 (0.4) | 3,763 (1.9) |
| High infant birth weight (≥4,000 g) | | |  |  |  |  |  |  |
| No | 15,141 (89.2) | 372,008 (95.5) | 414,276 (94.8) | 1,184,598 (92.0) | 13,433 (88.4) | 1,272,816 (89.6) | 81,115 (91.7) | 180,065 (90.5) |
| Yes | 1,715 (10.1) | 16,799 (4.3) | 19,582 (4.5) | 100,961 (7.8) | 1,719 (11.3) | 140,018 (9.9) | 7,019 (7.9) | 15,568 (7.8) |
| Unknown | 116 (0.7) | 785 (0.2) | 3,319 (0.8) | 2,657 (0.2) | 45 (0.3) | 7,790 (0.5) | 334 (0.4) | 3,281 (1.6) |

AIAN, American Indian or Alaskan Native; BMI, body mass index; GED, general educational development; NHOPI, Native Hawaiian or Other Pacific Islander; SVD, spontaneous vaginal delivery.

^*^Values are displayed as column totals and percentages; n (%).

# **Appendix S3. Demographic and clinical characteristics of primiparous individuals, United States, 2016-2021 (N=5,634,219)^*^**

|  | AIAN  (n=38,906) | Asian  (n=366,148) | Black  (n=756,993) | Hispanic  (n=1,147,275) | NHOPI  (n=10,299) | White  (n=3,183,618) | Multiple Race  (n=130,980) |
| --- | --- | --- | --- | --- | --- | --- | --- |
| Patient demographics | |  |  |  |  |  |  |
| Age (years) |  |  |  |  |  |  |  |
| ≤ 19 | 10,662 (27.4) | 5,299 (1.4) | 146,739 (19.4) | 237,103 (20.7) | 1,806 (17.5) | 261,336 (8.2) | 25,570 (19.5) |
| 20-24 | 16,013 (41.2) | 37,647 (10.3) | 286,564 (37.9) | 411,647 (35.9) | 4,454 (43.2) | 785,653 (24.7) | 47,301 (36.1) |
| 25-29 | 7,642 (19.6) | 123,566 (33.7) | 176,908 (23.4) | 271,046 (23.6) | 2,401 (23.3) | 1,014,966 (31.9) | 30,569 (23.3) |
| 30-34 | 3,262 (8.4) | 142,538 (38.9) | 97,297 (12.9) | 156,418 (13.6) | 1,173 (11.4) | 821,423 (25.8) | 19,492 (14.9) |
| 35-40 | 1,143 (2.9) | 48,049 (13.1) | 39,841 (5.3) | 58,889 (5.1) | 367 (3.6) | 257,969 (8.1) | 6,867 (5.2) |
| > 40 | 184 (0.5) | 9,049 (2.5) | 9,644 (1.3) | 12,172 (1.1) | 98 (1.0) | 42,271 (1.3) | 1,181 (0.9) |
| Marital Status |  |  |  |  |  |  |  |
| Married | 8,864 (22.8) | 322,741 (88.1) | 171,324 (22.6) | 441,607 (38.5) | 3,706 (36.0) | 2,125,826 (66.8) | 50,634 (38.7) |
| Unmarried | 30,042 (77.2) | 43,407 (11.9) | 585,669 (77.4) | 705,668 (61.5) | 6,593 (64.0) | 1,057,792 (33.2) | 80,346 (61.3) |
| Education |  |  |  |  |  |  |  |
| 8^th^ grade or less | 474 (1.2) | 4,217 (1.2) | 7,697 (1.0) | 47,252 (4.1) | 216 (2.1) | 25,896 (0.8) | 1,092 (0.8) |
| High school, N.D | 8,337 (21.4) | 12,466 (3.4) | 94,132 (12.4) | 192,035 (16.7) | 2,048 (19.9) | 173,458 (5.4) | 16,717 (12.8) |
| High school, or GED | 14,361 (36.9) | 37,119 (10.1) | 271,031 (35.8) | 380,598 (33.2) | 3,989 (38.7) | 644,803 (20.3) | 37,478 (28.6) |
| College, N.D | 9,161 (23.5) | 32,537 (8.9) | 182,888 (24.2) | 236,408 (20.6) | 2,288 (22.2) | 557,024 (17.5) | 31,882 (24.3) |
| Associate | 2,475 (6.4) | 20,537 (5.6) | 48,318 (6.4) | 78,716 (6.9) | 724 (7.0) | 284,796 (8.9) | 8,817 (6.7) |
| Bachelor’s | 2,965 (7.6) | 132,322 (36.1) | 100,691 (13.3) | 149,556 (13.0) | 803 (7.8) | 935,942 (29.4) | 22,536 (17.2) |
| Masters | 862 (2.2) | 92,483 (25.3) | 42,129 (5.6) | 48,964 (4.3) | 186 (1.8) | 435,401 (13.7) | 9,211 (7.0) |
| Doctorate | 271 (0.7) | 34,467 (9.4) | 10,107 (1.3) | 13,746 (1.2) | 45 (0.4) | 126,298 (4.0) | 3,247 (2.5) |
| Payer |  |  |  |  |  |  |  |
| Medicaid | 24,451 (62.8) | 76,076 (20.8) | 466,673 (61.6) | 635,488 (55.4) | 5,727 (55.6) | 797,031 (25.0) | 60,125 (45.9) |
| Private insurance | 9,332 (24.0) | 266,527 (72.8) | 242,907 (32.1) | 384,504 (33.5) | 2,910 (28.3) | 2,206,753 (69.3) | 60,706 (46.3) |
| Self-pay | 661 (1.7) | 13,357 (3.6) | 21,135 (2.8) | 79,624 (6.9) | 600 (5.8) | 71,356 (2.2) | 2,478 (1.9) |
| Other | 4,462 (11.5) | 10,188 (2.8) | 26,278 (3.5) | 47,659 (4.2) | 1,062 (10.3) | 108,478 (3.4) | 7,671 (5.9) |
| WIC | 21,790 (56.0) | 62,765 (17.1) | 424,970 (56.1) | 586,737 (51.1) | 4,231 (41.1) | 698,734 (21.9) | 54,290 (41.4) |
| Nativity |  |  |  |  |  |  |  |
| US born | 38,423 (98.8) | 66,595 (18.2) | 642,361 (84.9) | 685,664 (59.8) | 3,852 (37.4) | 2,991,917 (94.0) | 119,827 (91.5) |
| Foreign born | 483 (1.2) | 299,553 (81.8) | 114,632 (15.1) | 461,611 (40.2) | 6,447 (62.6) | 191,701 (6.0) | 11,153 (8.5) |
| Clinical characteristics | |  |  |  |  |  |  |
| Mode of delivery |  |  |  |  |  |  |  |
| SVD | 27,297 (70.2) | 222,872 (60.9) | 478,234 (63.2) | 778,481 (67.9) | 6,609 (64.2) | 2,099,463 (65.9) | 89,463 (68.3) |
| Forceps | 300 (0.8) | 5,044 (1.4) | 4,867 (0.6) | 8,278 (0.7) | 144 (1.4) | 34,985 (1.1) | 1,175 (0.9) |
| Vacuum | 1,423 (3.7) | 24,829 (6.8) | 26,662 (3.5) | 40,651 (3.5) | 415 (4.0) | 145,530 (4.6) | 5,242 (4.0) |
| Cesarean delivery with trial of labor | 6,442 (16.6) | 61,452 (16.8) | 136,618 (18.0) | 172,150 (15.0) | 1,821 (17.7) | 512,887 (16.1) | 21,156 (16.2) |
| Cesarean delivery without trial of labor | 3,444 (8.9) | 51,951 (14.2) | 110,612 (14.6) | 147,715 (12.9) | 1,310 (12.7) | 390753 (12.3) | 13,944 (10.6) |
| Pre-pregnancy BMI kg/m^2^ | |  |  |  |  |  |  |
| Underweight (< 18.5) | 1,202 (3.1) | 30,876 (8.4) | 32,367 (4.3) | 44,210 (3.9) | 256 (2.5) | 111,096 (3.5) | 5,740 (4.4) |
| Normal weight (18.5-24.9) | 14,608 (37.5) | 231,627 (63.3) | 291,214 (38.5) | 504,848 (44.0) | 3,334 (32.4) | 1,530,155 (48.1) | 57,970 (44.3) |
| Overweight (25.0-29.9) | 10,236 (26.3) | 75,015 (20.5) | 193,182 (25.5) | 317,323 (27.7) | 2,847 (27.6) | 788,549 (24.8) | 32,333 (24.7) |
| Obese (≥ 30.0) | 12,860 (33.1) | 28,630 (7.8) | 240,230 (31.7) | 280,894 (24.5) | 3,862 (37.5) | 753,818 (23.7) | 34,937 (26.7) |
| Assisted reproductive  technology | 73 (0.2) | 9,031 (2.5) | 3,665 (0.5) | 5,604 (0.5) | 27 (0.3) | 50,998 (1.6) | 1,107 (0.8) |
| Pre-pregnancy diabetes | 737 (1.9) | 2,945 (0.8) | 7,570 (1.0) | 8,824 (0.8) | 150 (1.5) | 22,784 (0.7) | 1,105 (0.8) |
| Pre-pregnancy hypertension | 925 (2.4) | 3,391 (0.9) | 21,622 (2.9) | 12,950 (1.1) | 170 (1.7) | 61,052 (1.9) | 2,653 (2.0) |
| Gestational diabetes | 3,166 (8.1) | 43,777 (12.0) | 32,386 (4.3) | 60,021 (5.2) | 742 (7.2) | 172,362 (5.4) | 6,963 (5.3) |
| Preeclampsia/eclampsia | 4,883 (12.6) | 19,271 (5.3) | 86,008 (11.4) | 93,728 (8.2) | 1,153 (11.2) | 338,900 (10.6) | 13,398 (10.2) |
| Induction of labour | 14,524 (37.3) | 112,326 (30.7) | 260,467 (34.4) | 371,565 (32.4) | 2,792 (27.1) | 1,230,312 (38.6) | 47,858 (36.5) |
| Augmentation of labour | 11,868 (30.5) | 118,248 (32.3) | 190,835 (25.2) | 338,895 (29.5) | 3,381 (32.8) | 888,127 (27.9) | 38,775 (29.6) |
| Gestational age (weeks) |  |  |  |  |  |  |  |
| Pre-term (<37) | 4,615 (11.9) | 31,297 (8.5) | 105,410 (13.9) | 117,396 (10.2) | 1,504 (14.6) | 273,170 (8.6) | 13,062 (10.0) |
| Term (37-41) | 31,392 (80.7) | 317,182 (86.6) | 611,427 (80.8) | 957,221 (83.4) | 7,942 (77.1) | 2,712,597 (85.2) | 109,533 (83.6) |
| Post-term (≥42) | 2,899 (7.5) | 17,669 (4.8) | 40,156 (5.3) | 72,658 (6.3) | 853 (8.3) | 197,851 (6.2) | 8,385 (6.4) |
| High infant birth weight  (≥4,000 g) | 3,214 (8.3) | 11,782 (3.2) | 25,176 (3.3) | 58,924 (5.1) | 631 (6.1) | 241,922 (7.6) | 7,774 (5.9) |

AIAN, American Indian or Alaskan Native; BMI, body mass index GED, general educational development; NHOPI, Native Hawaiian or Other Pacific Islander; SVD, spontaneous vaginal delivery.

^*^Values are displayed as column totals and percentages; n (%).

# **Appendix S4. Demographic and clinical characteristics of multiparous individuals without a previous cesarean delivery, United States, 2016-2021 (N=9,610,026)^*^**

|  | AIAN  (n=88,153) | Asian  (n=469,377) | Black  (n=1,468,188) | Hispanic  (n=2,120,835) | NHOPI  (n=23,082) | White  (n=5,229,044) | Multiple Race  (n=211,347) |
| --- | --- | --- | --- | --- | --- | --- | --- |
| Patient demographics | |  |  |  |  |  |  |
| Age (years) |  |  |  |  |  |  |  |
| ≤ 19 | 2,948 (3.3) | 1,337 (0.3) | 38,440 (2.6) | 59,512 (2.8) | 412 (1.8) | 64,245 (1.2) | 6,780 (3.2) |
| 20-24 | 22,021 (25.0) | 24,175 (5.2) | 322,917 (22.0) | 450,345 (21.2) | 5,115 (22.2) | 746,151 (14.3) | 52,320 (24.8) |
| 25-29 | 29,974 (34.0) | 98,829 (21.1) | 475,757 (32.4) | 658,519 (31.0) | 7,708 (33.4) | 1,534,399 (29.3) | 67,225 (31.8) |
| 30-34 | 21,353 (24.2) | 192,811 (41.1) | 376,568 (25.6) | 555,866 (26.2) | 6,059 (26.2) | 1,801,940 (34.5) | 52,269 (24.7) |
| 35-40 | 9,834 (11.2) | 124,150 (26.4) | 201,551 (13.7) | 313,420 (14.8) | 3,045 (13.2) | 905,800 (17.3) | 26,794 (12.7) |
| > 40 | 2,023 (2.3) | 28,075 (6.0) | 52,955 (3.6) | 83,173 (3.9) | 743 (3.2) | 176,509 (3.4) | 5,959 (2.8) |
| Marital Status |  |  |  |  |  |  |  |
| Married | 29,578 (33.6) | 408,780 (87.1) | 468,838 (31.9) | 1,060,092 (50.0) | 12,039 (52.2) | 3,840,173 (73.4) | 100,995 (47.8) |
| Unmarried | 58,575 (66.4) | 60,597 (12.9) | 999,350 (68.1) | 1,060,743 (50.0) | 11,043 (47.8) | 1,388,871 (26.6) | 110,352 (52.2) |
| Education |  |  |  |  |  |  |  |
| 8^th^ grade or less | 1,170 (1.3) | 17,925 (3.8) | 25,210 (1.7) | 230,460 (10.9) | 738 (3.2) | 89,213 (1.7) | 2,451 (1.2) |
| High school, N.D | 16,671 (18.9) | 26,102 (5.6) | 162,203 (11.0) | 391,887 (18.5) | 5,058 (21.9) | 306,067 (5.9) | 22,678 (10.7) |
| High school, or GED | 32,843 (37.3) | 66,691 (14.2) | 537,175 (36.6) | 697,579 (32.9) | 8,509 (36.9) | 1,160,403 (22.2) | 59,203 (28.0) |
| College, N.D | 22,958 (26.0) | 51,027 (10.9) | 373,801 (25.5) | 392,038 (18.5) | 5,375 (23.3) | 1,036,269 (19.8) | 58,855 (27.8) |
| Associate | 6,947 (7.9) | 30,615 (6.5) | 121,401 (8.3) | 139,180 (6.6) | 1,562 (6.8) | 523,131 (10.0) | 19,117 (9.0) |
| Bachelor’s | 5,458 (6.2) | 149,187 (31.8) | 163,729 (11.2) | 193,571 (9.1) | 1,421 (6.2) | 1,330,185 (25.4) | 31,880 (15.1) |
| Masters | 1,706 (1.9) | 90,880 (19.4) | 70,058 (4.8) | 60,570 (2.9) | 351 (1.5) | 615,883 (11.8) | 13,007 (6.2) |
| Doctorate | 400 (0.5) | 36,950 (7.9) | 1,4611 (1.0) | 15,550 (0.7) | 68 (0.3) | 167,893 (3.2) | 4,156 (2.0) |
| Payer |  |  |  |  |  |  |  |
| Medicaid | 60,306 (68.4) | 136,072 (29.0) | 980,719 (66.8) | 1,283,303 (60.5) | 13,749 (59.6) | 1,664,060 (31.8) | 109,623 (51.9) |
| Private insurance | 16,804 (19.1) | 304,635 (64.9) | 403,189 (27.5) | 571,275 (26.9) | 5,776 (25.0) | 3,178,764 (60.8) | 84,882 (40.2) |
| Self-pay | 1,679 (1.9) | 15,389 (3.3) | 40,472 (2.8) | 182,340 (8.6) | 1,603 (6.9) | 210,706 (4.0) | 5,228 (2.5) |
| Other | 9,364 (10.6) | 1,3281 (2.8) | 43,808 (3.0) | 83,917 (4.0) | 1,954 (8.5) | 175,514 (3.4) | 11,614 (5.5) |
| WIC | 46,026 (52.2) | 108,603 (23.1) | 764,189 (52.0) | 1,117,899 (52.7) | 9,274 (40.2) | 1,215,696 (23.2) | 81,307 (38.5) |
| Nativity |  |  |  |  |  |  |  |
| US born | 87,228 (99.0) | 90,160 (19.2) | 1,212,285 (82.6) | 1,055,173 (49.8) | 7,727 (33.5) | 4,914,301 (94.0) | 191,191 (90.5) |
| Foreign born | 925 (1.0) | 379,217 (80.8) | 255,903 (17.4) | 1,065,662 (50.2) | 15,355 (66.5) | 314,743 (6.0) | 20,156 (9.5) |
| Clinical characteristics | |  |  |  |  |  |  |
| Mode of delivery |  |  |  |  |  |  |  |
| SVD | 74,854 (84.9) | 376,032 (80.1) | 1,154,009 (78.6) | 1,758,499 (82.9) | 19,056 (82.6) | 4,333,942 (82.9) | 174,862 (82.7) |
| Forceps | 237 (0.3) | 2,786 (0.6) | 5,453 (0.4) | 7,278 (0.3) | 124 (0.5) | 22,959 (0.4) | 915 (0.4) |
| Vacuum | 1,284 (1.5) | 15,272 (3.3) | 27,327 (1.9) | 33,828 (1.6) | 419 (1.8) | 104,445 (2.0) | 4,008 (1.9) |
| Cesarean delivery with trial of labor | 4,525 (5.1) | 28,583 (6.1) | 112,988 (7.7) | 112,081 (5.3) | 1,267 (5.5) | 280,729 (5.4) | 12,983 (6.1) |
| Cesarean delivery without trial of labor | 7,253 (8.2) | 46,704 (10.0) | 168,411 (11.5) | 209,149 (9.9) | 2,216 (9.6) | 486,969 (9.3) | 18,579 (8.8) |
| Pre-pregnancy BMI kg/m^2^ | |  |  |  |  |  |  |
| Underweight (< 18.5) | 1,707 (1.9) | 28,872 (6.2) | 41,366 (2.8) | 44,245 (2.1) | 336 (1.5) | 161,492 (3.1) | 6,757 (3.2) |
| Normal weight (18.5-24.9) | 25,949 (29.4) | 275,766 (58.8) | 464,647 (31.6) | 732,891 (34.6) | 5,287 (22.9) | 2,414,775 (46.2) | 82,371 (39.0) |
| Overweight (25.0-29.9) | 24,824 (28.2) | 115,413 (24.6) | 407,978 (27.8) | 672,725 (31.7) | 6,177 (26.8) | 1,341,507 (25.7) | 56,346 (26.7) |
| Obese (≥ 30.0) | 35,673 (40.5) | 49,326 (10.5) | 554,197 (37.7) | 670,974 (31.6) | 11,282 (48.9) | 1,311,270 (25.1) | 65,873 (31.2) |
| Assisted reproductive  technology | 104 (0.1) | 8,592 (1.8) | 4,479 (0.3) | 6,566 (0.3) | 41 (0.2) | 59,828 (1.1) | 1,393 (0.7) |
| Pre-pregnancy diabetes | 2,006 (2.3) | 4,848 (1.0) | 16,901 (1.2) | 22,369 (1.1) | 376 (1.6) | 33,377 (0.6) | 1,765 (0.8) |
| Pre-pregnancy hypertension | 2,411 (2.7) | 5,287 (1.1) | 61,286 (4.2) | 29,747 (1.4) | 362 (1.6) | 96,965 (1.9) | 4,943 (2.3) |
| Gestational diabetes | 9,662 (11.0) | 65,385 (13.9) | 83,357 (5.7) | 169,628 (8.0) | 2,211 (9.6) | 322,914 (6.2) | 14,230 (6.7) |
| Preeclampsia/eclampsia | 7,335 (8.3) | 19,198 (4.1) | 119,944 (8.2) | 116,677 (5.5) | 1,626 (7.0) | 350,882 (6.7) | 14,764 (7.0) |
| Induction of labour | 29,672 (33.7) | 125,576 (26.8) | 450,454 (30.7) | 614,794 (29.0) | 5,458 (23.6) | 1,908,065 (36.5) | 70,734 (33.5) |
| Augmentation of labour | 21,835 (24.8) | 123,573 (26.3) | 334,469 (22.8) | 523,553 (24.7) | 6,425 (27.8) | 1,220,365 (23.3) | 53,765 (25.4) |
| Gestational age (weeks) |  |  |  |  |  |  |  |
| Pre-term (<37) | 11,492 (13.0) | 41,387 (8.8) | 220,780 (15.0) | 229,291 (10.8) | 3,456 (15.0) | 426,468 (8.2) | 22,218 (10.5) |
| Term (37-41) | 70,971 (80.5) | 407,497 (86.8) | 1,174,257 (80.0) | 1,768,368 (83.4) | 17,811 (77.2) | 4,523,847 (86.5) | 176,517 (83.5) |
| Post-term (≥42) | 5,690 (6.5) | 20,493 (4.4) | 73,151 (5.0) | 123,176 (5.8) | 1,815 (7.9) | 278,729 (5.3) | 12,612 (6.0) |
| High infant birth weight  (≥4,000 g) | 9,678 (11.0) | 20,439 (4.4) | 65,347 (4.5) | 151,353 (7.1) | 2,287 (9.9) | 541,596 (10.4) | 16,725 (7.9) |

AIAN, American Indian or Alaskan Native; BMI, body mass index; GED, general educational development; NHOPI, Native Hawaiian or Other Pacific Islander; SVD, spontaneous vaginal delivery.

^*^Values are displayed as column totals and percentages; n (%).

# **Appendix S5. Demographic and clinical characteristics of multiparous individuals with a previous cesarean delivery, United States, 2016-2021 (N=2,762,563)^*^**

|  | AIAN  (n=23,253) | Asian  (n=151,498) | Black  (n=467,706) | Hispanic  (n=651,357) | NHOPI  (n=7,105) | White  (n=1,407,132) | Multiple Race  (n=54,512) |
| --- | --- | --- | --- | --- | --- | --- | --- |
| Patient demographics | |  |  |  |  |  |  |
| Age (years) |  |  |  |  |  |  |  |
| ≤ 19 | 368 (1.6) | 95 (0.1) | 4,691 (1.0) | 7,274 (1.1) | 60 (0.8) | 6,504 (0.5) | 659 (1.2) |
| 20-24 | 4,153 (17.9) | 3,509 (2.3) | 68,982 (14.7) | 93,337 (14.3) | 1,145 (16.1) | 134,884 (9.6) | 9,689 (17.8) |
| 25-29 | 7,699 (33.1) | 23,004 (15.2) | 144,655 (30.9) | 191,627 (29.4) | 2,162 (30.4) | 360,846 (25.6) | 17,042 (31.3) |
| 30-34 | 6,755 (29.1) | 61,954 (40.9) | 140,477 (30.0) | 197,226 (30.3) | 2,215 (31.2) | 517,210 (36.8) | 15,747 (28.9) |
| 35-40 | 3,465 (14.9) | 50,545 (33.4) | 85,573 (18.3) | 127,058 (19.5) | 1,213 (17.1) | 319,375 (22.7) | 9,170 (16.8) |
| > 40 | 813 (3.5) | 12,391 (8.2) | 23,328 (5.0) | 34,835 (5.3) | 310 (4.4) | 68,313 (4.9) | 2,205 (4.0) |
| Marital Status |  |  |  |  |  |  |  |
| Married | 8,336 (35.8) | 139,063 (91.8) | 172,997 (37.0) | 355,714 (54.6) | 3,849 (54.2) | 1,057,901 (75.2) | 27,452 (50.4) |
| Unmarried | 14,917 (64.2) | 12,435 (8.2) | 294,709 (63.0) | 295,643 (45.4) | 3,256 (45.8) | 349,231 (24.8) | 27,060 (49.6) |
| Education |  |  |  |  |  |  |  |
| 8^th^ grade or less | 319 (1.4) | 4,951 (3.3) | 7,403 (1.6) | 65,844 (10.1) | 224 (3.2) | 16,642 (1.2) | 597 (1.1) |
| High school, N.D | 4,257 (18.3) | 8,040 (5.3) | 50,117 (10.7) | 114,634 (17.6) | 1,517 (21.4) | 78,652 (5.6) | 5,549 (10.2) |
| High school, or GED | 8,549 (36.8) | 19,918 (13.1) | 163,093 (34.9) | 208,077 (31.9) | 2,647 (37.3) | 303,126 (21.5) | 14,957 (27.4) |
| College, N.D | 6,036 (26.0) | 14,288 (9.4) | 118,511 (25.3) | 121,860 (18.7) | 1,638 (23.1) | 283,003 (20.1) | 15,331 (28.1) |
| Associate | 1,956 (8.4) | 9,187 (6.1) | 42,744 (9.1) | 44,862 (6.9) | 477 (6.7) | 147,360 (10.5) | 5,170 (9.5) |
| Bachelor’s | 1,542 (6.6) | 51,029 (33.7) | 54,583 (11.7) | 68,413 (10.5) | 463 (6.5) | 355,563 (25.3) | 8,298 (15.2) |
| Masters | 474 (2.0) | 33,014 (21.8) | 25,719 (5.5) | 21,758 (3.3) | 118 (1.7) | 176,912 (12.6) | 3,473 (6.4) |
| Doctorate | 120 (0.5) | 11,071 (7.3) | 5,536 (1.2) | 5,909 (0.9) | 21 (0.3) | 45,874 (3.3) | 1,137 (2.1) |
| Payer |  |  |  |  |  |  |  |
| Medicaid | 15,911 (68.4) | 42,411 (28.0) | 311,295 (66.6) | 394,541 (60.6) | 4,346 (61.2) | 457,087 (32.5) | 29,436 (54.0) |
| Private insurance | 4,695 (20.2) | 100,352 (66.2) | 132,146 (28.3) | 180,550 (27.7) | 1,827 (25.7) | 877,352 (62.4) | 21,519 (39.5) |
| Self-pay | 354 (1.5) | 5,206 (3.4) | 10,760 (2.3) | 49,634 (7.6) | 450 (6.3) | 30,184 (2.1) | 919 (1.7) |
| Other | 2,293 (9.9) | 3,529 (2.3) | 13,505 (2.9) | 26,632 (4.1) | 482 (6.8) | 42,509 (3.0) | 2,638 (4.8) |
| WIC | 12,407 (53.4) | 32,736 (21.6) | 24,4085 (52.2) | 350,932 (53.9) | 2,987 (42.0) | 338,936 (24.1) | 21,983 (40.3) |
| Nativity |  |  |  |  |  |  |  |
| US born | 22,985 (98.8) | 20,890 (13.8) | 377,066 (80.6) | 298,785 (45.9) | 2,179 (30.7) | 1,317,417 (93.6) | 48,835 (89.6) |
| Foreign born | 268 (1.2) | 130,608 (86.2) | 90,640 (19.4) | 352,572 (54.1) | 4,926 (69.3) | 89,715 (6.4) | 5,677 (10.4) |
| Clinical characteristics | |  |  |  |  |  |  |
| Mode of delivery |  |  |  |  |  |  |  |
| Spontaneous vaginal | 3,280 (14.1) | 19,909 (13.1) | 59,403 (12.7) | 81,181 (12.5) | 1,377 (19.4) | 184,837 (13.1) | 7,575 (13.9) |
| Forceps | 22 (0.1) | 446 (0.3) | 625 (0.1) | 866 (0.1) | 25 (0.4) | 2,395 (0.2) | 102 (0.2) |
| Vacuum | 104 (0.4) | 2,027 (1.3) | 3,080 (0.7) | 4,057 (0.6) | 86 (1.2) | 9,885 (0.7) | 348 (0.6) |
| Cesarean delivery with trial of labor | 1,119 (4.8) | 10,461 (6.9) | 33,586 (7.2) | 35,421 (5.4) | 481 (6.8) | 62,854 (4.5) | 2,926 (5.4) |
| Cesarean delivery without trial of labor | 18,728 (80.5) | 118,655 (78.3) | 371,012 (79.3) | 529,832 (81.3) | 5,136 (72.3) | 1,147,161 (81.5) | 43,561 (79.9) |
| Pre-pregnancy BMI kg/m^2^ | |  |  |  |  |  |  |
| Underweight (< 18.5) | 247 (1.1) | 6,088 (4.0) | 6,963 (1.5) | 8,483 (1.3) | 54 (0.8) | 26,185 (1.9) | 963 (1.8) |
| Normal weight (18.5-24.9) | 4,728 (20.3) | 78,554 (51.9) | 104,700 (22.4) | 179,105 (27.5) | 1,152 (16.2) | 498,553 (35.4) | 15,794 (29.0) |
| Overweight (25.0-29.9) | 5,952 (25.6) | 44,620 (29.5) | 121,769 (26.0) | 201,407 (30.9) | 1,705 (24.0) | 367,460 (26.1) | 13,985 (25.7) |
| Obese (≥ 30.0) | 12,326 (53.0) | 22,236 (14.7) | 234,274 (50.1) | 262,362 (40.3) | 4,194 (59.0) | 514,934 (36.6) | 23,770 (43.6) |
| Assisted reproductive  technology | 50 (0.2) | 2,656 (1.8) | 1,474 (0.3) | 2,189 (0.3) | 7 (0.1) | 19,387 (1.4) | 378 (0.7) |
| Pre-pregnancy diabetes | 1,173 (5.0) | 2,703 (1.8) | 10,567 (2.3) | 13,283 (2.0) | 245 (3.4) | 21,039 (1.5) | 1,096 (2.0) |
| Pre-pregnancy hypertension | 1,005 (4.3) | 2,711 (1.8) | 30,108 (6.4) | 14,691 (2.3) | 209 (2.9) | 43,146 (3.1) | 2,073 (3.8) |
| Gestational diabetes | 3,032 (13.0) | 25,293 (16.7) | 36,707 (7.8) | 67,168 (10.3) | 911 (12.8) | 116,055 (8.2) | 4,939 (9.1) |
| Preeclampsia/eclampsia | 2,247 (9.7) | 6,618 (4.4) | 42,281 (9.0) | 40,612 (6.2) | 625 (8.8) | 101,954 (7.2) | 4,129 (7.6) |
| Induction of labour | 1,545 (6.6) | 8,404 (5.5) | 32,066 (6.9) | 34,671 (5.3) | 500 (7.0) | 78,181 (5.6) | 3,376 (6.2) |
| Augmentation of labour | 1,471 (6.3) | 11,246 (7.4) | 29,057 (6.2) | 39,569 (6.1) | 806 (11.3) | 79,539 (5.7) | 3,670 (6.7) |
| Gestational age (weeks) |  |  |  |  |  |  |  |
| Pre-term (<37) | 3,929 (16.9) | 15,626 (10.3) | 80,343 (17.2) | 86,361 (13.3) | 1,326 (18.7) | 147,488 (10.5) | 7,126 (13.1) |
| Term (37-41) | 18,061 (77.7) | 130,605 (86.2) | 367,407 (78.6) | 534,025 (82.0) | 5,274 (74.2) | 1,200,031 (85.3) | 44,709 (82.0) |
| Post-term (≥42) | 1,263 (5.4) | 5,267 (3.5) | 19,956 (4.3) | 30,971 (4.8) | 505 (7.1) | 59,613 (4.2) | 2,677 (4.9) |
| High infant birth weight  (≥4,000 g) | 2,958 (12.7) | 8,905 (5.9) | 27,516 (5.9) | 56,143 (8.6) | 837 (11.8) | 167,267 (11.9) | 4,988 (9.2) |

AIAN, American Indian or Alaskan Native; BMI, body mass index; GED, general educational development; NHOPI, Native Hawaiian or Other Pacific Islander; SVD, spontaneous vaginal delivery.

^*^Values are displayed as column totals and percentages; n (%).

# **Appendix S6. Rate of obstetric anal sphincter injury (%) by race and ethnicity in primiparous and multiparous individuals, United States 2016-2021**

|  | Spontaneous Vaginal | | | Forceps | | | Vacuum | | | Total | | |
| --- | --- | --- | --- | --- | --- | --- | --- | --- | --- | --- | --- | --- |
|  | No. deliveries | No. cases | Rate (%) | No. deliveries | No. cases | Rate (%) | No. deliveries | No. cases | Rate (%) | No. deliveries | No. cases | Rate (%) |
| Primiparous (N=4,001,964) | | |  |  |  |  |  |  |  |  |  |  |
| Total | 3,702,419 | 63,804 | 1.7 | 54,793 | 8,094 | 14.8 | 244,752 | 16,267 | 6.6 | 4,001,964 | 88,165 | 2.2 |
| AIAN | 27,297 | 515 | 1.9 | 300 | 36 | 12.0 | 1,423 | 81 | 5.7 | 29,020 | 632 | 2.2 |
| Asian | 222,872 | 7,283 | 3.3 | 5,044 | 1,109 | 22.0 | 24,829 | 2,713 | 10.9 | 252,745 | 11,105 | 4.4 |
| Black | 478,234 | 4,711 | 1.0 | 4,867 | 574 | 11.8 | 26,662 | 1,091 | 4.1 | 509,763 | 6,376 | 1.3 |
| Hispanic | 778,481 | 10,047 | 1.3 | 8,278 | 901 | 10.9 | 40,651 | 2,010 | 4.9 | 827,410 | 12,958 | 1.6 |
| NHOPI | 6,609 | 135 | 2.0 | 144 | 8 | 5.6 | 415 | 25 | 6.0 | 7,168 | 168 | 2.3 |
| White | 2,099,463 | 39,906 | 1.9 | 34,985 | 5,320 | 15.2 | 145,530 | 10,073 | 6.9 | 2,279,978 | 55,299 | 2.4 |
| Multiple race | 89,463 | 1,207 | 1.3 | 1,175 | 146 | 12.4 | 5,242 | 274 | 5.2 | 95,880 | 1,627 | 1.7 |
| Multiparous without a previous cesarean delivery (N=8,117,589) | | | | | | |  |  |  |  |  |  |
| Total | 7,891,254 | 37,895 | 0.5 | 39,752 | 2,997 | 7.5 | 185,583 | 5,908 | 3.2 | 8,117,589 | 46,800 | 0.6 |
| AIAN | 74,854 | 195 | 0.3 | 237 | 8 | 3.4 | 1,284 | 40 | 3.1 | 76,375 | 243 | 0.3 |
| Asian | 376,032 | 4,136 | 1.1 | 2,786 | 380 | 13.6 | 15,272 | 999 | 6.5 | 394,090 | 5,515 | 1.4 |
| Black | 1,154,009 | 3,129 | 0.3 | 5,453 | 256 | 4.7 | 27,327 | 478 | 1.7 | 1,186,789 | 3,863 | 0.3 |
| Hispanic | 1,758,499 | 6,374 | 0.4 | 7,278 | 342 | 4.7 | 32,828 | 661 | 2.0 | 1,798,605 | 7,377 | 0.4 |
| NHOPI | 19,056 | 71 | 0.4 | 124 | 3 | 2.4 | 419 | 8 | 1.9 | 19,599 | 82 | 0.4 |
| White | 4,333,942 | 23,305 | 0.5 | 22,959 | 1,947 | 8.5 | 104,445 | 3,608 | 3.5 | 4,461,346 | 28,860 | 0.6 |
| Multiple race | 174,862 | 685 | 0.4 | 915 | 61 | 6.7 | 4,008 | 114 | 2.8 | 179,785 | 860 | 0.5 |
| Multiparous with a previous cesarean delivery (N=381,630) | | | | | |  |  |  |  |  |  |  |
| Total | 357,562 | 4,724 | 1.3 | 4,481 | 527 | 11.8 | 19,587 | 1,001 | 5.1 | 381,630 | 6,252 | 1.6 |
| AIAN | 3,280 | 20 | 0.6 | 22 | 3 | 13.6 | 104 | 1 | 1.0 | 3,406 | 24 | 0.7 |
| Asian | 19,909 | 624 | 3.1 | 446 | 75 | 16.8 | 2,027 | 188 | 9.3 | 22,382 | 887 | 4.0 |
| Black | 59,403 | 418 | 0.7 | 625 | 61 | 9.8 | 3,080 | 82 | 2.7 | 63,108 | 561 | 0.9 |
| Hispanic | 81,181 | 676 | 0.8 | 866 | 65 | 7.5 | 4,057 | 150 | 3.7 | 86,104 | 891 | 1.0 |
| NHOPI | 1,377 | 15 | 1.1 | 25 | 2 | 8.0 | 86 | 4 | 4.7 | 1,488 | 21 | 1.4 |
| White | 184,837 | 2,887 | 1.6 | 2,395 | 308 | 12.9 | 9,885 | 562 | 5.7 | 197,117 | 3,757 | 1.9 |
| Multiple race | 7,575 | 84 | 1.1 | 102 | 13 | 12.7 | 348 | 14 | 4.0 | 8,025 | 111 | 1.4 |

AIAN, American Indian or Alaskan Native; NHOPI, Native Hawaiian or Other Pacific Islander

# **Appendix S7. Rate of obstetric anal sphincter injury (%) by race and ethnicity in multiparous individuals with at least one previous cesarean delivery and one prior birth (sensitivity analysis), United States 2016-2021 (N=114,540)**

|  | Spontaneous Vaginal | | | Forceps | | | Vacuum | | |
| --- | --- | --- | --- | --- | --- | --- | --- | --- | --- |
|  | No. deliveries | No. cases | Rate (%) | No. deliveries | No.  cases | Rate (%) | No. deliveries | No. cases | Rate (%) |
| Multiparous with a previous cesarean | | | |  |  |  |  |  |  |
| AIAN | 637 | 15 | 2.4 | 8 | 1 | 12.5 | 24 | 0 | 0 |
| Asian | 8,855 | 408 | 4.6 | 270 | 49 | 18.1 | 1,133 | 134 | 11.8 |
| Black | 12,951 | 201 | 1.6 | 220 | 26 | 11.8 | 983 | 41 | 4.2 |
| Hispanic | 23,728 | 368 | 1.6 | 386 | 39 | 10.1 | 1,683 | 93 | 5.5 |
| NHOPI | 257 | 11 | 4.3 | 7 | 1 | 14.3 | 32 | 3 | 9.4 |
| White | 55,644 | 1,584 | 2.8 | 1,142 | 181 | 15.8 | 4,364 | 322 | 7.4 |
| Multiple race | 2,037 | 45 | 2.2 | 40 | 4 | 10.0 | 139 | 6 | 4.3 |

AIAN, American Indian or Alaskan Native; NHOPI, Native Hawaiian or Other Pacific Islander

# **Appendix S8. Crude and adjusted hazard ratios (HRs) and 95% confidence intervals (CIs) for obstetric anal sphincter injury by mode of delivery, among multiparous individuals with at least one previous cesarean delivery and one prior birth (sensitivity analysis), United States, 2016-2021 (N=114,540)^*^**

|  | Crude Rate (%) | | HR  (95% CI) | | aHR  (95% CI) |
| --- | --- | --- | --- | --- | --- |
| Spontaneous Vaginal | |  | |  |  |
| AIAN | 2.4 | | 0.91 (0.54, 1.51) | | 1.43 (0.86, 2.38) |
| Asian | 4.6 | | **1.93 (1.74, 2.16)** | | **1.70 (1.52, 1.91)** |
| Black | 1.6 | | **0.64 (0.55, 0.74)** | | 0.92 (0.79, 1.08) |
| Hispanic | 1.6 | | **0.61 (0.55, 0.69)** | | **0.80 (0.71, 0.91)** |
| NHOPI | 4.3 | | 1.63 (0.89, 2.94) | | 2.70 (1.48, 4.90) |
| White | 2.8 | | Ref | | Ref |
| Multiple race | 2.2 | | **0.82 (0.61, 1.10)** | | 1.01 (0.75, 1.36) |
| Forceps |  | |  | |  |
| AIAN | 12.5 | | 1.38 (0.19, 9.86) | | 2.95 (0.39, 21.98) |
| Asian | 18.1 | | 1.25 (0.90, 1.71) | | 1.05 (0.76, 1.46) |
| Black | 11.8 | | 0.88 (0.58, 1.32) | | 1.09 (0.69, 1.71) |
| Hispanic | 10.1 | | 0.72 (0.51, 1.02) | | 0.89 (0.60, 1.32) |
| NHOPI | 14.3 | | 1.34 (0.19, 9.61) | | 1.83 (0.23, 14.18) |
| White | 15.8 | | Ref | | Ref |
| Multiple race | 10.0 | | 0.72 (0.27, 1.93) | | 0.83 (0.30, 2.26) |
| Vacuum |  | |  | |  |
| AIAN | 0 | | **-** | | - |
| Asian | 11.8 | | **1.95 (1.59, 2.39)** | | **1.69 (1.37, 2.09)** |
| Black | 4.2 | | 0.62 (0.45, 0.86) | | 0.78 (0.55, 1.10) |
| Hispanic | 5.5 | | 0.80 (0.64, 1.01) | | 0.92 (0.71, 1.19) |
| NHOPI | 9.4 | | 1.28 (0.41, 4.00) | | 1.76 (0.56, 5.57) |
| White | 7.4 | | Ref | | Ref |
| Multiple race | 4.3 | | 0.64 (0.28, 1.43) | | 0.66 (0.29, 1.47) |

AIAN, American Indian or Alaskan Native; NHOPI, Native Hawaiian or Other Pacific Islander

Bold text indicates statistically significant hazard ratios.

*Adjusted for maternal age, marital status, education, payer, WIC, pre-pregnancy body mass index, assisted reproductive technology, pre-pregnancy diabetes, pre-pregnancy hypertension, gestational diabetes, preeclampsia/eclampsia, induction of labour, augmentation of labour, high infant birth weight (≥4,000g).

# **Appendix S9. Crude and adjusted hazard ratios (HRs) and 95% confidence intervals (CIs) for obstetric anal sphincter injury by race and ethnicity and mode of delivery, among primiparous and multiparous individuals using multiple imputation for missing data (sensitivity analysis), United States, 2016-2021^*^**

|  | Primiparous | | | Multiparous without previous CD | | | Multiparous with previous CD | | |
| --- | --- | --- | --- | --- | --- | --- | --- | --- | --- |
|  | Crude  Rate (%) | HR  (95% CI) | aHR  (95% CI) | Crude  Rate (%) | HR  (95% CI) | aHR  (95% CI) | Crude Rate (%) | HR  (95% CI) | aHR  (95% CI) |
| Spontaneous |  |  |  |  |  |  |  |  |  |
| AIAN | 1.9 | **1.06 (1.02, 1.10)** | **1.44 (1.39, 1.49)** | 0.3 | **0.53 (0.49, 0.57)** | **0.73 (0.68, 0.79)** | 0.6 | **0.47 (0.41, 0.54)** | **0.81 (0.71, 0.92)** |
| Asian | 3.3 | **1.89 (1.88, 1.91)** | **1.66 (1.64, 1.67)** | 1.1 | **2.19 (2.16, 2.23)** | **2.00 (1.97, 2.04)** | 3.1 | **2.43 (2.37, 2.49)** | **2.18 (2.13, 2.24)** |
| Black | 1.0 | **0.63 (0.63, 0.64)** | **0.91 (0.90, 0.92)** | 0.3 | **0.61 (0.59, 0.62)** | **0.89 (0.87, 0.91)** | 0.7 | **0.55 (0.54, 0.57)** | **0.90 (0.87, 0.93)** |
| Hispanic | 1.3 | **0.72 (0.71, 0.72)** | **0.94 (0.93, 0.95)** | 0.4 | **0.72 (0.71, 0.73)** | **1.06 (1.04, 1.07)** | 0.8 | **0.63 (0.61, 0.64)** | **0.96 (0.94, 0.99)** |
| NHOPI | 2.0 | **1.16 (1.09, 1.23)** | **1.51 (1.42, 1.60)** | 0.4 | **0.74 (0.66, 0.82)** | **0.90 (0.86, 0.93)** | 1.1 | **0.71 (0.61, 0.82)** | **1.21 (1.05, 1.40)** |
| White | 1.9 | Ref | Ref | 0.5 | Ref | Ref | 1.6 | Ref | Ref |
| Multiple race | 1.3 | **0.75 (0.74, 0.77)** | **0.91 (0.89, 0.93)** | 0.4 | **0.75 (0.72, 0.73)** | **0.90 (0.86, 0.91)** | 1.1 | **0.78 (0.73, 0.83)** | 1.00 (0.94, 1.06) |
| Forceps |  |  |  |  |  |  |  |  |  |
| AIAN | 12.0 | **0.85 (0.75, 0.97)** | 1.04 (0.92, 1.18) | 3.4 | **0.54 (0.39, 0.75)** | **0.68 (0.49, 0.95)** | 13.6 | **1.61 (1.22, 2.13)** | **1.89 (1.40, 2.53)** |
| Asian | 22.0 | **1.66 (1.62, 1.70)** | **1.52 (1.48, 1.56)** | 13.6 | **1.71 (1.62, 1.82)** | **1.50 (1.41, 1.59)** | 16.8 | **1.45 (1.35, 1.56)** | **1.30 (1.21, 1.40)** |
| Black | 11.8 | **0.91 (0.88, 0.94)** | **1.13 (1.10, 1.18)** | 4.7 | **0.63 (0.59, 0.68)** | **0.88 (0.82, 0.95)** | 9.8 | **0.87 (0.80, 0.95)** | **1.16 (1.06, 1.27)** |
| Hispanic | 10.9 | **0.73 (0.71, 0.75)** | **0.85 (0.83, 0.88)** | 4.7 | **0.55 (0.51, 0.58)** | **0.78 (0.73, 0.83)** | 7.5 | **0.57 (0.52, 0.61)** | **0.74 (0.68, 0.81)** |
| NHOPI | 5.6 | **0.55 (0.45, 0.67)** | **0.66 (0.54, 0.81)** | 2.4 | **0.45 (0.28, 0.72)** | 0.66 (0.41, 1.05) | 8.0 | **1.40 (1.02, 1.91)** | **1.65 (1.20, 2.26)** |
| White | 15.2 | Ref | Ref | 8.5 | Ref | Ref | 12.9 | Ref | Ref |
| Multiple race | 12.4 | **0.82 (0.77, 0.88)** | **0.89 (0.84, 0.95)** | 6.7 | **0.83 (0.73, 0.95)** | 0.95 (0.83, 1.08) | 12.7 | 1.03 (0.88, 1.21) | **1.28 (1.08, 1.51)** |
| Vacuum |  |  |  |  |  |  |  |  |  |
| AIAN | 5.7 | **0.91 (0.84, 0.99)** | **1.24 (1.14, 1.35)** | 3.1 | 0.97 (0.82, 1.15) | **1.38 (1.15, 1.64)** | 1.0 | **0.17 (0.09, 0.32)** | **0.24 (0.13, 0.45)** |
| Asian | 10.9 | **1.76 (1.73, 1.78)** | **1.54 (1.52, 1.57)** | 6.5 | **2.04 (1.97, 2.11)** | **1.79 (1.73, 1.86)** | 9.3 | **1.88 (1.80, 1.97)** | **1.67 (1.59, 1.75)** |
| Black | 4.1 | **0.69 (0.67, 0.71)** | **0.95 (0.93, 0.97)** | 1.7 | **0.58 (0.55, 0.61)** | **0.83 (0.78, 0.87)** | 2.7 | **0.52 (0.48, 0.55)** | **0.70 (0.65, 0.75)** |
| Hispanic | 4.9 | **0.75 (0.74, 0.76)** | **0.93 (0.91, 0.95)** | 2.0 | **0.60 (0.57, 0.62)** | **0.82 (0.79, 0.86)** | 3.7 | **0.64 (0.60, 0.67)** | **0.82 (0.78, 0.87)** |
| NHOPI | 6.0 | **0.93 (0.81, 1.07)** | **1.27 (1.10, 1.46)** | 1.9 | **0.62 (0.44, 0.87)** | 0.92 (0.84, 1.03) | 4.7 | 0.97 (0.73, 1.28) | 1.29 (0.97, 1.70) |
| White | 6.9 | Ref | Ref | 3.5 | Ref | Ref | 5.7 | Ref | Ref |
| Multiple race | 5.2 | **0.78 (0.74, 0.81)** | **0.90 (0.86, 0.94)** | 2.8 | **0.80 (0.72, 0.88)** | 0.93 (0.84, 1.03) | 4.0 | **0.77 (0.67, 0.90)** | **0.85 (0.74, 0.99)** |

AIAN, American Indian or Alaskan Native; NHOPI, Native Hawaiian or Other Pacific Islander.

Bold text indicates statistically significant adjusted hazard ratios (aHRs).

*Adjusted for maternal age, marital status, education, payer, WIC, pre-pregnancy body mass index, assisted reproductive technology, pre-pregnancy diabetes, pre-pregnancy hypertension, gestational diabetes, preeclampsia/eclampsia, induction of labour, augmentation of labour, high infant birth weight (≥4,000g).

# **Appendix S10. Bias analysis correcting for outcome misclassification and unmeasured confounding for obstetric anal sphincter injury by race and ethnicity and mode of delivery, among primiparous and multiparous individuals (sensitivity analysis), United States, 2016-2021**

**
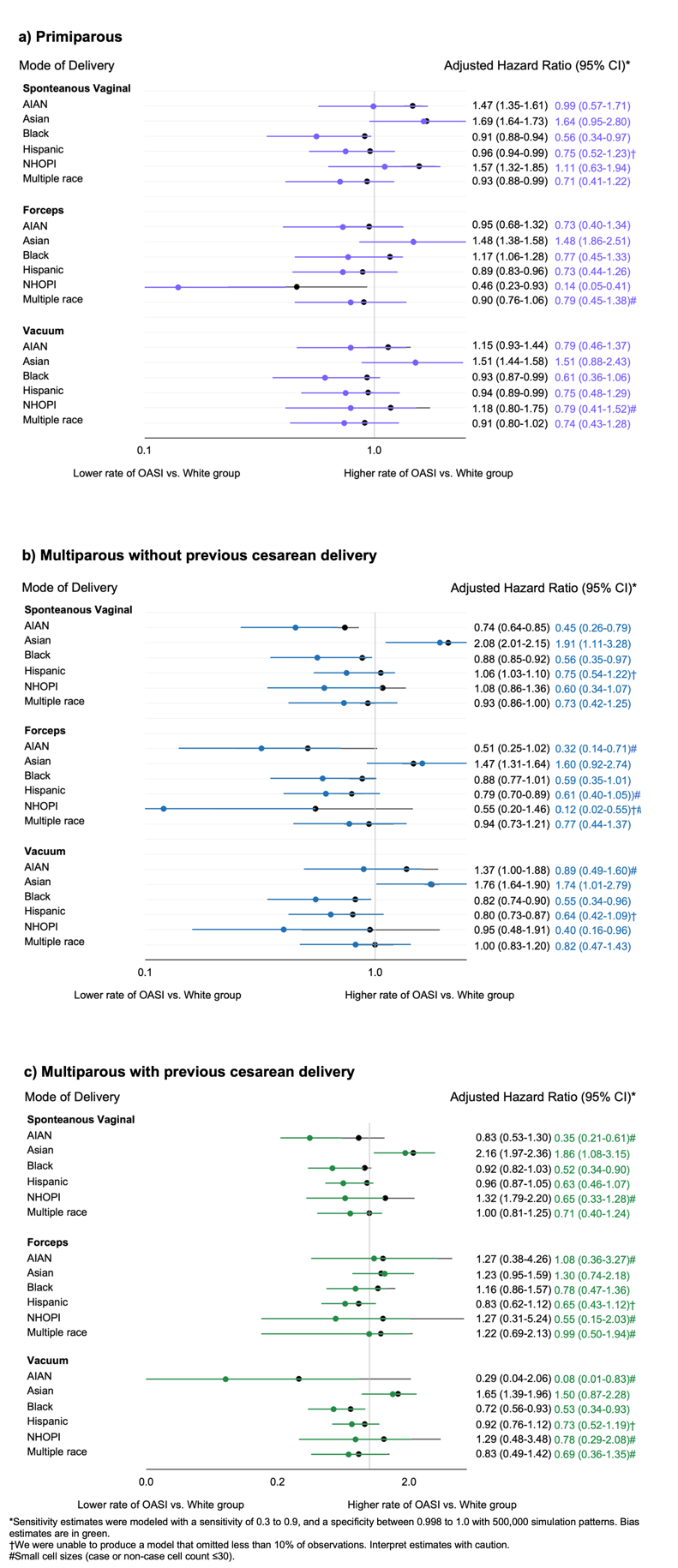
**

# **Appendix S11. Rates of obstetric anal sphincter injury by Asian race subcategories among spontaneous vaginal, forceps, and vacuum delivery, stratified by parity and obstetric history, United States, 2016-2021**

|  | Spontaneous Vaginal | | | Forceps | | | Vacuum | | |
| --- | --- | --- | --- | --- | --- | --- | --- | --- | --- |
|  | No. deliveries | No. cases | Rate (%) | No. deliveries | No. cases | Rate (%) | No. deliveries | No. cases | Rate (%) |
| Primiparous (N=2,532,723) | | |  |  |  |  |  |  |  |
| Chinese | 51,569 | 1,518 | 2.9 | 967 | 186 | 19.2 | 5,368 | 489 | 9.1 |
| Filipino | 21,540 | 579 | 2.7 | 476 | 70 | 14.7 | 2,074 | 179 | 8.6 |
| Indian | 71,814 | 2,993 | 4.2 | 1,863 | 507 | 27.2 | 9,448 | 1,234 | 13.1 |
| Japanese | 6,041 | 136 | 2.3 | 182 | 17 | 9.3 | 455 | 36 | 7.9 |
| Korean | 13,480 | 318 | 2.4 | 342 | 73 | 21.3 | 1,605 | 126 | 7.9 |
| Vietnamese | 18,884 | 598 | 3.2 | 349 | 64 | 18.3 | 1,866 | 470 | 9.6 |
| White | 2,099,463 | 39,906 | 1.9 | 34,985 | 5,320 | 15.2 | 145,530 | 10,073 | 6.9 |
| Other Asian | 39,544 | 1,158 | 2.9 | 865 | 192 | 22.2 | 4,013 | 470 | 11.7 |
| Multiparous without a previous cesarean (N=4,855,436) | | | | |  |  |  |  |  |
| Chinese | 74,950 | 808 | 1.1 | 504 | 67 | 13.3 | 3,006 | 177 | 5.9 |
| Filipino | 40,603 | 351 | 0.9 | 330 | 32 | 9.7 | 1,622 | 87 | 5.4 |
| Indian | 106,331 | 1,649 | 1.6 | 944 | 176 | 18.6 | 5,466 | 452 | 8.3 |
| Japanese | 10,500 | 68 | 0.6 | 111 | 5 | 4.5 | 336 | 19 | 5.7 |
| Korean | 21,287 | 174 | 0.8 | 161 | 13 | 8.1 | 803 | 29 | 3.6 |
| Vietnamese | 31,152 | 318 | 1.0 | 193 | 21 | 10.9 | 1,119 | 65 | 5.8 |
| White | 4,333,942 | 23,305 | 0.5 | 22,959 | 1,947 | 8.5 | 104,445 | 3,608 | 3.5 |
| Other Asian | 91,209 | 768 | 0.8 | 543 | 66 | 12.2 | 2,920 | 170 | 5.8 |
| Multiparous with a previous cesarean (N=219,499) | | | | |  |  |  |  |  |
| Chinese | 3,108 | 89 | 2.9 | 56 | 11 | 19.6 | 292 | 28 | 9.6 |
| Filipino | 1,892 | 41 | 2.2 | 31 | 6 | 19.3 | 171 | 10 | 5.8 |
| Indian | 6,952 | 308 | 4.4 | 190 | 42 | 22.1 | 879 | 100 | 11.4 |
| Japanese | 421 | 17 | 4.0 | 20 | 3 | 15.0 | 38 | 1 | 2.6 |
| Korean | 923 | 23 | 2.5 | 25 | 4 | 16.0 | 85 | 5 | 5.9 |
| Vietnamese | 1,315 | 29 | 2.2 | 21 | 1 | 4.8 | 121 | 5 | 4.1 |
| White | 184,837 | 2,887 | 1.6 | 2,395 | 308 | 12.9 | 9,885 | 562 | 5.7 |
| Other Asian | 5,298 | 117 | 2.2 | 103 | 8 | 7.6 | 441 | 39 | 8.8 |

# **Appendix S12. Crude and adjusted hazard ratios (HRs) and 95% confidence intervals (CIs) for obstetric anal sphincter injury by Asian race subcategories and mode of delivery, stratified by parity and obstetric history (sensitivity analysis), United States, 2016-2021^*^**

|  | Primiparous (n=2,532,723) | | | Multiparous without previous CD (n=4,855,436) | | | Multiparous with previous CD (n = 219,499) | | |
| --- | --- | --- | --- | --- | --- | --- | --- | --- | --- |
|  | Crude  Rate (%) | HR  (95% CI) | aHR  (95% CI) | Crude  Rate (%) | HR  (95% CI) | aHR  (95% CI) | Crude Rate (%) | HR  (95% CI) | aHR  (95% CI) |
| Spontaneous |  |  |  |  |  |  |  |  |  |
| Chinese | 2.9 | **1.66 (1.58, 1.74)** | **1.37 (1.30, 1.44)** | 1.1 | **2.16 (2.02, 2.32)** | **1.92 (1.79, 2.06)** | 2.9 | **2.15 (1.74, 2.66)** | **1.87 (1.51, 2.32)** |
| Filipino | 2.7 | **1.80 (1.65, 1.95)** | **1.67 (1.54, 1.81)** | 0.9 | **1.97 (1.77, 2.18)** | **1.84 (1.66, 2.05)** | 2.2 | **1.77 (1.30, 2.41)** | **1.76 (1.29, 2.40)** |
| Indian | 4.2 | **2.58 (2.48, 2.67)** | **2.10 (2.02, 2.18)** | 1.6 | **3.31 (3.15, 3.48)** | **2.72 (2.58, 2.86)** | 4.4 | **3.47 (3.08, 3.90)** | **2.73 (2.42, 3.09)** |
| Japanese | 2.3 | **1.29 (1.02, 1.53)** | 1.07 (0.91, 1.27) | 0.6 | **1.35 (1.07, 1.71)** | 1.14 (0.90, 1.44) | 4.0 | **2.69 (1.67, 4.33)** | **2.34 (1.45, 3.78)** |
| Korean | 2.4 | **1.26 (1.13, 1.40)** | 1.00 (0.90, 1.12) | 0.8 | **1.56 (1.34, 1.81)** | **1.26 (1.08, 1.46)** | 2.5 | **1.87 (1.24, 2.82)** | 1.48 (0.98, 2.23) |
| Vietnamese | 3.2 | **2.03 (1.87, 2.02)** | **1.94 (1.79, 2.10)** | 1.0 | **2.23 (2.00, 2.49)** | **2.35 (2.10, 2.62)** | 2.2 | **1.75 (1.21, 2.52)** | **1.80 (1.25, 2.60)** |
| White | 1.9 | Ref | Ref | 0.5 | Ref | Ref | 1.6 | Ref | Ref |
| Other Asian | 2.9 | **1.75 (1.65, 1.85)** | **1.71 (1.61, 1.82)** | 0.8 | **1.65 (1.54, 1.77)** | **1.94 (1.81, 2.09)** | 2.2 | **1.62 (1.35, 1.95)** | **1.89 (1.57, 2.28)** |
| Forceps |  |  |  |  |  |  |  |  |  |
| Chinese | 19.2 | **1.32 (1.15, 1.53)** | **1.19 (1.03, 1.38)** | 13.3 | **1.57 (1.23, 2.00)** | 1.26 (0.98, 1.62) | 19.6 | 1.65 (0.90, 3.02) | 1.34 (0.73, 2.49) |
| Filipino | 14.7 | 1.16 (0.92, 1.47) | **1.08 (1.85, 1.37)** | 9.7 | 1.32 (0.93, 1.87) | 1.18 (0.83, 1.67) | 19.3 | 1.57 (0.70, 3.53) | 1.42 (0.62, 3.25) |
| Indian | 27.2 | **2.00 (1.82, 2.19)** | **1.82 (1.65, 2.00)** | 18.6 | **2.33 (2.00, 2.72)** | **1.88 (1.61, 2.21)** | 22.1 | **1.93 (1.40, 2.66)** | **1.62 (1.16, 2.26)** |
| Japanese | 9.3 | 0.64 (0.40, 1.03) | **0.66 (0.35, 0.91)** | 4.5 | 0.61 (0.26, 1.48) | 0.45 (0.19, 1.10) | 15.0 | 1.19 (0.38, 3.72) | 1.06 (0.33, 3.42) |
| Korean | 21.3 | **1.43 (1.13, 1.80)** | 1.24 (0.98, 1.57) | 8.1 | 0.92 (0.54, 1.50) | 0.78 (0.45, 1.34) | 16.0 | 1.46 (0.54, 3.91) | 0.96 (0.35, 2.63) |
| Vietnamese | 18.3 | **1.42 (1.11, 1.82)** | **1.42 (1.11, 1.82)** | 10.9 | 1.43 (0.93, 2.20) | 1.48 (0.96, 2.28) | 4.8 | 0.44 (0.06, 3.13) | 0.46 (0.06, 3.32) |
| White | 15.2 | Ref | Ref | 8.5 | Ref | Ref | 12.9 | Ref | Ref |
| Other Asian | 22.2 | **1.72 (1.50, 1.99)** | **1.75 (1.51, 2.02)** | 12.2 | **1.46 (1.15, 1.87)** | **1.64 (1.28, 2.11)** | 7.6 | 0.57 (0.28, 1.15) | 0.54 (0.26, 1.11) |
| Vacuum |  |  |  |  |  |  |  |  |  |
| Chinese | 9.1 | **1.39 (1.27, 1.52)** | **1.18 (1.07, 1.29)** | 5.9 | **1.84 (1.58, 2.14)** | **1.61 (1.38, 1.88)** | 9.6 | **1.94 (1.33, 2.83)** | **1.64 (1.11, 2.42)** |
| Filipino | 8.6 | **1.48 (1.28, 1.72)** | **1.33 (1.15, 1.55)** | 5.4 | **1.83 (1.48, 2.26)** | **1.62 (1.31, 2.00)** | 5.8 | 1.34 (0.72, 2.51) | 1.17 (0.62, 2.20) |
| Indian | 13.1 | **2.14 (2.02, 2.27)** | **1.79 (1.68, 1.90)** | 8.3 | **2.64 (2.39, 2.91)** | **2.07 (1.87, 2.29)** | 11.4 | **2.26 (1.83, 2.80)** | **1.93 (1.54, 2.41)** |
| Japanese | 7.9 | 1.22 (0.88, 1.69) | 0.97 (0.70, 1.35) | 5.7 | **1.64 (1.04, 2.57)** | 1.34 (0.85, 2.10) | 2.6 | 0.59 (0.08, 4.18) | 0.48 (0.07, 2.40) |
| Korean | 7.9 | 1.13 (0.95, 1.35) | 0.93 (0.78, 1.11) | 3.6 | 1.06 (0.73, 1.52) | 0.80 (0.56, 1.16) | 5.9 | 1.19 (0.49, 2.88) | 1.04 (0.43, 2.52) |
| Vietnamese | 9.6 | **1.56 (1.49, 1.64)** | **1.56 (1.34, 1.81)** | 5.8 | **1.80 (1.41, 2.30)** | **1.76 (1.38, 2.26)** | 4.1 | 0.97 (0.40, 2.33) | 0.89 (0.37, 2.17) |
| White | 6.9 | Ref | Ref | 3.5 | Ref | Ref | 5.7 | Ref | Ref |
| Other Asian | 11.7 | **1.67 (1.44, 1.94)** | **1.74 (1.59, 1.92)** | 5.8 | **1.77 (1.52, 2.06)** | **1.92 (1.64, 2.24)** | 8.8 | **1.72 (1.25, 2.38)** | **1.74 (1.24, 2.43)** |

AIAN, American Indian or Alaskan Native; NHOPI, Native Hawaiian or Other Pacific Islander.

Bold text indicates statistically significant hazard ratios (HRs).

*Adjusted for maternal age, marital status, education, payer, WIC, pre-pregnancy body mass index, assisted reproductive technology, pre-pregnancy diabetes, pre-pregnancy hypertension, gestational diabetes, preeclampsia/eclampsia, induction of labour, augmentation of labour, high infant birth weight (≥4,000g).
